# Uncovering Microbiome Adaptations in a Full-Scale Biogas Plant: Insights from MAG-Centric Metagenomics and Metaproteomics

**DOI:** 10.3390/microorganisms11102412

**Published:** 2023-09-27

**Authors:** Julia Hassa, Tom Jonas Tubbesing, Irena Maus, Robert Heyer, Dirk Benndorf, Mathias Effenberger, Christian Henke, Benedikt Osterholz, Michael Beckstette, Alfred Pühler, Alexander Sczyrba, Andreas Schlüter

**Affiliations:** 1 Genome Research of Industrial Microorganisms, Center for Biotechnology (CeBiTec), Bielefeld University, Universitätsstrasse 27, 33615 Bielefeld, Germany; jhassa@cebitec.uni-bielefeld.de (J.H.);; 2 Computational Metagenomics Group, Center for Biotechnology (CeBiTec), Bielefeld University, Universitätsstraße 27, 33615 Bielefeld, Germany; t.tubbesing@uni-bielefeld.de (T.J.T.);; 3 Multidimensional Omics Data Analyses Group, Leibniz-Institut für Analytische Wissenschaften-ISAS-e.V., Bunsen-Kirchhoff-Straße 11, Dortmund 44139, Germany; 4 Multidimensional Omics Data Analyses Group, Faculty of Technology, Bielefeld University, Universitätsstraße 25, 33615 Bielefeld, Germany; 5 Biosciences and Process Engineering, Anhalt University of Applied Sciences, Bernburger Straße 55, Postfach 1458, 06366 Köthen, Germany; 6 Bioprocess Engineering, Otto von Guericke University, Universitätsplatz 2, 39106 Magdeburg, Germany; 7 Bioprocess Engineering, Max Planck Institute for Dynamics of Complex Technical Systems, Sandtorstraße 1, 39106 Magdeburg, Germany; 8 Bavarian State Research Center for Agriculture, Institute for Agricultural Engineering and Animal Husbandry, Vöttinger Straße 36, 85354 Freising, Germany

**Keywords:** biogas microbiome, biogas process chain, anaerobic digestion, metagenomic binning, metagenome analyses, metaproteome analyses

## Abstract

The current focus on renewable energy in global policy highlights the importance of methane production from biomass through anaerobic digestion (AD). To improve biomass digestion while ensuring overall process stability, microbiome-based management strategies become more important. In this study, metagenomes and metaproteomes were used for metagenomically assembled genome (MAG)-centric analyses to investigate a full-scale biogas plant consisting of three differentially operated digesters. Microbial communities were analyzed regarding their taxonomic composition, functional potential, as well as functions expressed on the proteome level. Different abundances of genes and enzymes related to the biogas process could be mostly attributed to different process parameters. Individual MAGs exhibiting different abundances in the digesters were studied in detail, and their roles in the hydrolysis, acidogenesis and acetogenesis steps of anaerobic digestion could be assigned. *Methanoculleus thermohydrogenotrophicum* was an active hydrogenotrophic methanogen in all three digesters, whereas *Methanothermobacter wolfeii* was more prevalent at higher process temperatures. Further analysis focused on MAGs, which were abundant in all digesters, indicating their potential to ensure biogas process stability. The most prevalent MAG belonged to the class *Limnochordia*; this MAG was ubiquitous in all three digesters and exhibited activity in numerous pathways related to different steps of AD.

## 1. Introduction

Great efforts are currently being made to promote the energy transition towards an increased share of renewable energies in the energy supply. One of the most versatile renewable energy carriers is biogas from anaerobic digestion (AD), which still is and will be an important renewable energy source in Germany and numerous other countries. In the future, however, biogas plants will have to become much more flexible in terms of their operating strategies and feedstocks (e.g., biowaste, residues from animal husbandry and food production), as their use for flexible heat and power generation is becoming increasingly important for the energy transition. Under such conditions, given the complexity and inertia of the AD process, it will be more difficult to maintain stable conditions and achieve adequate biogas yields to be economically profitable. Therefore, robust operating strategies for biogas plants must be developed based not only on process parameters but also on the microbial communities within biogas digesters. Complex biogas microbiomes are responsible for the AD process, and certain members of these microbiomes are involved in the four AD phases: hydrolysis, acido-, aceto- and methanogenesis. To enable microbial community-based process management strategies, an improved knowledge of biogas microbiomes regarding their compositions and also their functions in the biogas process is essential. In this context, the roles of taxa adapted and specialized to different process conditions and also resilient taxa, which are process condition-independent and thus were suggested to support stable process conditions in biogas digesters (e.g., [1,2,3]), are of interest.

Since cultivation-based approaches are limited regarding the identification of the yet uncultivable fraction of biogas microbiomes and marker gene-based taxonomic analyses cannot give insights into the functional potential and the actually expressed functions of microbial communities, metagenome, metatranscriptome and metaproteome approaches have become important to gain insights into the yet uncultivable ’dark matter’ of anaerobic digestion [4,5].

Several studies have been published, where metagenomically assembled bacterial and archaeal genomes were reconstructed from biogas metagenomes. Parks et al. [6] assembled nearly 8000 MAGs from diverse metagenomes, which included also metagenomes from biogas digesters, leading to the reconstruction of numerous biogas-related MAGs. Campanaro et al. [7] analyzed biogas microbiomes from 12 mesophilic and thermophilic full-scale biogas plants and identified 132 MAGs for which a functional role in the AD process was predicted based on their genomic potential. Moreover, Campanaro et al. [8] metagenomically assembled nearly 1600 MAGs from 134 biogas metagenomes and described that biogas microbiomes are highly flexible and adaptable to different process conditions, including varying temperatures and substrates.

Combined metagenome and metatranscriptome analyses are less frequently performed for the analysis of biogas microbiomes (e.g., [9,10,11,12]) but yield useful insight into the transcriptional activity of whole microbial communities or single MAGs. Metatranscriptomics is also used in combination with genome analyses of isolates to elucidate their role in the biogas process (e.g., [13,14]). Similarly, metaproteome studies are rather rare in the biogas field (e.g., [15,16,17,18,19]), although these provide important insights into the actually expressed functions of microbial communities as well as single MAGs or isolates.

However, knowledge of microbial adaptations and, in particular, the different metabolic solutions performed by individual biogas microbiomes from differently operated digesters is still limited. With more profound insights into the ’dark matter’ of AD microbiomes based on their metagenomes as well as metabolic activity based on metaproteomes, microbiome-based process control and monitoring would be feasible. This could enable the development of knowledge-based operational strategies to better adapt biogas microbiomes to the expected or prevailing process conditions and to maintain the efficiency of the biogas process under varying conditions.

Besides the need for deeper metagenome- and metaproteome-based analyses of biogas plants, the processing of such datasets using bioinformatic tools and workflows poses a particular challenge. While large-scale studies investigating hundreds of metagenome datasets offer valuable insights, some research questions require a more in-depth investigation, focusing on the comparison of individual samples and the role of specific species found therein. Different approaches exist for the comparative analysis of metagenome and -proteome datasets, with different methods in terms of assembling and binning genomes [20,21], calculating the abundances of MAGs and genes [22], handling replicates, and creating functional profiles. Since meta-omics data are noisy, error-prone and the experimental setups differ, the choice of bioinformatic methods demands careful consideration, and methods are continually refined and expanded upon.

In a recent study, we analyzed the microbial taxonomic composition within 67 full-scale German biogas digesters, which featured stable process conditions, although they were operated differently [23]. The majority of the microbial community profiles determined for these biogas plants clustered in three different groups, which could be characterized by the corresponding process parameters and the specific taxa present. However, outlier samples were also identified, which clustered separately from the three main clusters. This separate clustering could most likely be linked to the differences in process parameters and operation (e.g., higher amounts of manure, higher process temperatures), and the correspondingly altered microbial communities in these plants. The analysis of these outlier microbiomes revealed a higher proportion of yet unknown *Actinobacteria* and *Proteobacteria*, which correlated with higher amounts of animal manure as substrate. To gain deeper insights into these outlier microbiomes, biogas plant 35 (BP35, [23]), consisting of three differentially operated digesters, was chosen as a representative plant of this outlier group for a combined deep metagenome and metaproteome analysis. The goal was to reconstruct MAGs to identify specific taxa, their genetic potential and actually expressed metabolic functions, in response to the different process conditions of the three differentially operated digesters. Moreover, resilient taxa and their metabolic activities should also be uncovered. These taxa might support stable process conditions within the three digesters of this biogas plant.

It is hypothesized that (i) the metagenome-based functional potential is much broader than the proteome-based actually expressed functions of the whole microbial community, (ii) specialized taxa are adapted to different process conditions (e.g., process temperatures, feedstocks) of the digesters, (iii) taxa for specific functions in the three digesters are represented by specific and different microbiome members to serve the biogas process chain, and (iv) taxa which are independent of the process operation show even abundances and constant metabolic activities within all digesters, indicating their important role for a stable process.

In this study, different biogas-related key genes and enzymes of the whole microbial community were considered to investigate the adaptation to different conditions in the three biogas digesters. In addition, MAGs were studied to identify those that are specific to one of the digesters and those that have high abundance in all digesters. Individual MAGs were analyzed in detail to elucidate their role in the biogas process.

## 2. Materials and Methods

### 2.1. Sampling at the Full-Scale Biogas Plant and Metadata Compilation

Sampling and metadata compilation for BP35 located in Eastern Bavaria (Germany) were performed as described by Hassa et al. [23]. Briefly, three replicates of 50 mL samples were collected from the main digesters after a three-month period of stable process conditions (February 2017). The samples were stored at −20 °C until microbiological, chemical, metagenomic and metaproteomic analyses were performed. Chemical analyses were performed as described previously [24]. Information regarding the process operation and condition was provided by the owner of the biogas plant and is shown in the Supplementary Table S1.

### 2.2. DNA Isolation, Metagenome Library Preparation and Sequencing

DNA was extracted in triplicate from the same 50 mL sample using the FastDNA${}^{®}$ Spin Kit for Soil (MP Biomedicals GmbH, Eschwege, Germany) in combination with the Genomic DNA Clean & Concentrator^TM^ purification kit (Zymo Research, Irvine, USA) according to the manufacturer’s instructions. Metagenomic sequence libraries were constructed in triplicate for each DNA triplicate according to the “TruSeq DNA PCR-free” protocol (Illumina Inc., San Diego, CA, USA) and subsequently sequenced on the Illumina HiSeq platform (2 × 250 bp, paired-end mode, two lanes per sample).

### 2.3. Bioinformatic Processing, Single-Read Analyses and Assembly/Binning of the Metagenomic Datasets

The raw sequencing reads were quality trimmed, and the forward and reverse reads of each triplicate were merged (Trimmomaticv 0.38; [25]). Hereby, the paired-end mode was used, TruSeq3 adapters were removed, and reads shorter than 100 bp were discarded. After quality control (FastQC; [26]) and the subsampling of each dataset to one million reads (seqtk; [27]), single-read analyses were performed, applying the MGX platform [28]. These analyses included the taxonomic classification (Kraken/Diamond/RefSeq) and identification of clusters of orthologous groups (COGs) of proteins. The resulting read counts for each analysis, in triplicate, were used to calculate median values, which were then normalized. Pearson correlations of the process and chemical analysis parameters with the twelve most abundant genera in triplicate were calculated and visualized as clustered heatmap with R.

Metagenome assembly and genome reconstruction by binning were performed by applying workflows implemented in the EMGB platform (Elastic MetaGenome Browser [5]). Processing of the data includes the assembly of metagenomic reads to contigs (MEGAHIT v1.1.3; [29]), as well as the prediction of coding regions on all assembled contigs (Prodigal v2.6.3; [30]). MAGs were reconstructed (MetaBAT v2.12.1; [31]) and taxonomically classified (GTDB-Tk v2.1.1; [32]) using the Genome Taxonomy Database (GTDB release 207; [33]). The number of reads which mapped onto individual genes was extracted from sequence alignment maps (Samtools v1.14; [34]; featureCounts; [35]). Functional annotations for genes were obtained (DIAMOND v2.0.5.143; [36]) using the KEGG database (release 02.2021; [37]). Annotations were discarded if the sequence identity was below 30% or the e-value was larger than 1 × 10${}^{-10}$. Read counts were summed up on the level of KEGG orthologies (KOs). Based on the resulting KO counts, average genomic copy numbers (AGCNs) were calculated and inter- as well as intra-sample normalization of the AGCNs was performed (MUSiCC v1.0.3; [38]). The AGCN of a genetic feature represents how many copies of a certain feature are found, on average, on a genome in the microbial community. The calculation takes into account that genomes in the community have different abundances.

### 2.4. Generation, Processing and Analyses of the Metaproteomic Datasets

The BP35 sample preparation for metaproteome analysis was carried out according to the protocol published by Heyer et al. [17]. Briefly, protein extraction and purification were performed three times for each sample of the three digesters using liquid phenol in a ball mill device. After solubilization in urea and protein quantification using an Amido Black reagent, proteins were separated by SDS-PAGE into ten fractions. Subsequently, proteins were digested within the gel and analyzed via nano liquid tandem mass spectrometry (LC-MS/MS) measurements using an UltiMate^®^ 3000 nano splitless reversed-phase nanoHPLC (Thermo Fisher Scientific, Dreieich, Germany) coupled online to a timsTOF^TM^ pro mass spectrometer (Bruker Daltonik GmbH, Bremen, Germany). The result files of the MS/MS measurement were processed (Compass DataAnalysis 5.1 software, Bruker Daltonik GmbH, Bremen) to create generic files for the subsequent protein identification (Mascot 2.5; [39]). The assembled and annotated contigs of the BP35 metagenome were used to identify metaproteins and, where possible, assign them to MAGs. Homologous, redundant protein identifications were grouped into metaproteins (protein groups) if they shared the same peptide set. Spectral counts of proteins which could not be uniquely assigned to one gene were excluded. Finally, a result matrix of all unique proteins for the three different digesters was exported. Spectral counts in each sample were total count normalized so that the sum of all spectral counts in each sample was equal (78,119 spectral counts). For each digester, median spectral counts were calculated for genes and KOs, and the results underwent a second round of total count normalization.

### 2.5. Metagenomically Assembled Genome (MAG)-Centric Metagenome and Metaproteome Analysis

The reconstruction of MAGs was performed by applying the EMGB platform (see Section 2.3), and the taxonomic classification was carried out using the Genome Taxonomy Database Toolkit (GTDB-Tk release 207; [33]). For MAGs that are described in detail, the GTDB species identifier is listed alongside the corresponding NCBI GenBank accession. In case a MAG is not classified on the species level, the lowest known taxonomic rank and the GenBank accession of the closest relative is provided. For the MAG-centric analyses, the completeness and contamination of the MAGs were estimated (CheckM v1.1.3; [40]), and their relative abundances in the three digesters were calculated based on read mappings (bbmap; [41]). When calculating the relative abundance of MAGs, the abundances of unbinned contigs were taken into account. Thus, the results show the MAG abundance as a fraction of the whole microbial community and not only the fraction of the successfully binned community. Unmapped reads (11–18%) were not considered during abundance estimation. For the MAG-centric metagenome analysis, all annotated genes and their relative abundances belonging to one MAG were used to identify the functional potential of a given MAG, with a focus on biogas related genes.

To identify the expressed functions (proteins), a MAG-centric metaproteome analysis was performed. For this purpose, all spectral counts of genes belonging to each MAG were summed up. The resulting numbers were adjusted by dividing by the respective MAG completeness value to prevent the underrepresentation of incomplete MAGs. The sum of the spectral counts assigned to unbinned contigs was also calculated and again, this allowed to calculate MAG proteomic abundance as a fraction of whole community proteomic abundance. The median relative proteome abundance for each digester was determined from the triplicate samples and then normalized so that the relative abundances of the unbinned contigs and all MAGs summed up to 100 percent. Logarithmic fold changes of KO-level spectral counts between samples were calculated as $log2FC=log_{2}\left(count_{x}+s/count_{y}+s\right)$, where count${}_{x}$ and count${}_{x}$ are the spectral counts for a KO in samples x and y. An offset of s = 2 (30-th percentile, rounded) was added to obtain sensible fold changes in cases where the spectral count was zero or close to zero in one of the samples. KEMET (v1.0.0; [42]) was used to calculate the completeness of KEGG modules in individual MAGs based on the metagenome. Only KEGG modules which were complete or missing one block were further considered. To assess the activity for each module in a MAG, the spectral counts of proteins involved in the module were divided through the total sum of all spectral counts originating from the MAG. Moreover, the expression of the biogas process-related key enzymes [43] was investigated to identify the role of certain MAGs in the biogas process.

## 3. Results and Discussion

### 3.1. The Full-Scale Biogas Plant 35 Consists of Three Parallel Lines Differing in the General Process Operation, Especially Feedstocks and Process Temperature

**Figure 1:**
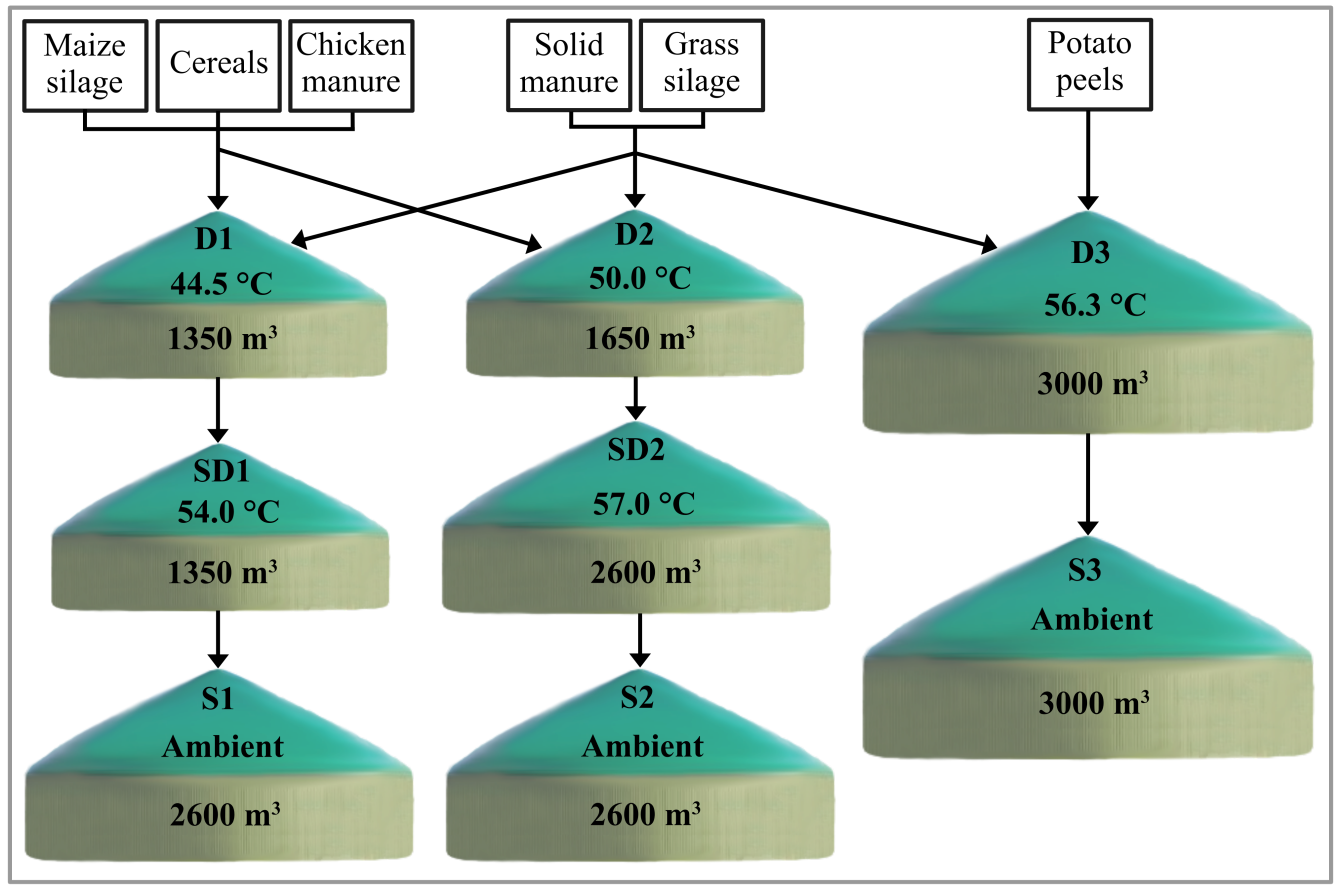
Schematic visualization of biogas plant 35 consisting of three separate lines. Indicated are the temperatures and volumes of the digesters. D1 = digester 1, SD1 = secondary digester 1, S1 = digestate storage 1; D2 = digester 2, SD2 = secondary digester 2, S2 = digestate storage 2; D3 = digester 3, S3 = digestate storage 3.

Biogas plant 35, located in Eastern Bavaria (Germany), belongs to a set of 67 stable running biogas digesters which were already taxonomically analyzed applying a 16S rRNA gene amplicon sequencing approach [23]. BP35 consists of three primary digesters (D1–D3), two heated secondary digesters (SD1, SD2) and three storage tanks at ambient temperatures (S1–S3), which are organized in three parallel lines (Figure 1). The three digesters and the secondary digesters are continuous stirred tank reactors (CSTRs), the main digesters feature reactor volumes of 1350 m${}^{3}$ (D1), 1650 m${}^{3}$ and 3000 m${}^{3}$. The complete biogas plant has a total installed electrical capacity of 1785 kW, producing about 9 GWh electricity per year, of which 3% is used for internal consumption. Moreover, 7.4 GWh of heat is produced per year, of which 1.3 GWh is used for heating the digesters and secondary digesters of this biogas plant. However, the share of the individual digesters in the total energy production of this biogas plant remains unknown, as the respective digesters do not have separate measuring points for the amount and quality of the produced biogas.

The three primary digesters were operated at different average temperature levels (D1: 44.5 °C, D2: 50.0 °C, D3: 56.3 °C) and also differed regarding their feed mix (Figure 1, Table 1). While D1 and D2 were fed mainly with maize and grass silage and solid manure from cattle and sheep, in combination with lower amounts of cereals and chicken manure, D3 was fed with grass silage in combination with high amounts of solid manure from cattle and sheep, potato peels and, occasionally, horse manure. The three digesters also featured differences in their general process characteristics and operation (Table 1 and Table S1).

The two digesters D1 and D2 were operated with approximately 20.5 t fresh matter (FM) m${}^{-3}$d${}^{-1}$, an organic loading rate (OLR) of approximately 4.4 kg${}_{\mathrm{VS}}$m${}^{-3}$d${}^{-1}$, and a hydraulic retention time of 73 days. In comparison, digester D3 was fed with 6.3 t FM m${}^{-3}$d${}^{-1}$, an OLR of 0.4 kg${}_{\mathrm{VS}}$ m${}^{-3}$d${}^{-1}$, and a retention time of 475 days. The total Kjeldahl nitrogen (TKN) values differed between samples from digester 1 and 2 (6.9 gL${}^{-1}$) compared to digester 3 (4.2 gL${}^{-1}$) and there were also considerable variations for total ammonium nitrogen (TAN) values (D1: 3.7 gL${}^{-1}$, D2: 4.3 gL${}^{-1}$, D3: 2.8 gL${}^{-1}$) and ammonia concentrations (D1: 65.1 mgL${}^{-1}$, D2: 157.5 mgL${}^{-1}$, D3: 95.8 mgL${}^{-1}$). The results from all chemical analyses are available in the Supplementary Table S1. As process parameters varied considerably between the three digesters, it is expected that different microbiomes developed within these digesters. In particular, microbial community compositions, the genetic potential, and the metaproteome profile should differentiate the digesters.

### 3.2. Taxonomic Profiling and Functional Potential Based on Metagenome Single-Read Analyses

To obtain an overview of the taxonomic profiles as well as the general functional potential of the microbiomes of the three BP35 digesters, single-read analyses based on the metagenomic datasets were carried out. Although this approach has its drawbacks, including reliance on read length and the completeness of the database used for sequence read classification, it offers the advantage of avoiding assembly or binning biases, allowing for comprehensive taxonomic and functional profiling of the entire community using the same sequence dataset.

#### 3.2.1. Differentially Abundant and Evenly Distributed Taxa Residing in the Three Digesters as Revealed by Taxonomic Profiling Based on Metagenome Single-Read Analyses

**Figure 2:**
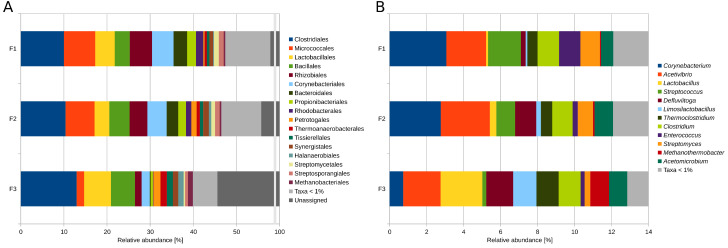
Normalized taxonomic profiles of the three digesters (D1–D3) of biogas plant 35 based on metagenomic single-read analyses. (**A**) Taxonomic profile of the most abundant taxa on order level, 42%, 44% and 54% of the reads remain unassigned for D1, D2 and D3, respectively. (**B**) Taxonomic profile of the most abundant taxa on genus level, 30%, 29% and 23% of the assigned taxa are below 1% relative abundance (marked in gray); 58%, 60% and 65% of the reads remain unassigned for D1, D2 and D3, respectively.

To deduce the taxonomic profiles of the microbiomes residing in digesters D1, D2 and D3 of BP35, single-read analyses applying the MGX platform were performed [28]. About 30%, 33% and 45% of the high-quality and subsampled reads were taxonomically assigned on the NCBI superkingdom level for D1, D2 and D3, respectively, and were used as input for the subsequent analyses. Based on the normalization of the reads assigned at the superkingdom level, the digesters D1 and D2 showed a similar proportion of *Bacteria* and *Archaea* of 98.9% vs. 1.1% and 98.6% vs. 1.4%, respectively, whereas D3 had with 97.5% vs. 2.5%, a much higher share of *Archaea*. The most abundant phyla in BP35 were *Firmicutes* (D1: 30.8%, D2: 34.4%, D3: 54.2%), *Actinobacteria* (D1: 24.4%, D2: 21.6%, D3: 7.8%), *Proteobacteria* (D1: 14.4%, D2: 11.6%, D3: 3.8%), *Bacteroidetes* (D1: 6.9%, D2: 5.6% and D3: 1.2%) and *Euryarchaeota* (D1: 1.0%, D2: 1.3%, D3: 2.4%). At the phylum level, about 20%, 21% and 27% of the input reads remained taxonomically unassigned for D1, D2 and D3, respectively. It became obvious that, besides the well-known phyla in biogas digesters, such as *Firmicutes* and *Bacteroidetes*, the phyla *Actinobacteria* and *Proteobacteria* had a higher share within this biogas plant compared to the taxonomic profiles of other biogas plants [23,44,45,46,47,48]. At the order level, members of the *Clostridiales* (D1: 10.0%, D2: 10.4%, D3: 13.0%), *Micrococcales* (D1: 7.2%, D2: 6.7%, D3: 1.7%), *Lactobacillales* (D1: 4.5%, D2: 3.4%, D3: 6.2%), *Bacillales* (D1: 3.5%, D2: 4.7%, D3: 5.6%), *Rhizobiales* (D1: 5.2%, D2: 4.1%, D3: 1.5%) and *Corynebacteriales* (D1: 5.0%, D2: 4.5%, D3: 1.9%) featured the highest relative abundances in the three digesters (Figure 2A).

At the genus level, members of the genera *Corynebacterium* (D1: 3.1%, D2: 2.8%, D3: 0.7%), *Streptococcus* (D1: 1.8%, D2: 1.0%, D3: 0.2%), *Enterococcus* (D1: 1.1%, D2: 0.3%, D3: 0.2%) and *Streptomyces* (D1: 1.1%, D2: 0.8%, D3: 0.3%) showed a decreasing relative abundance from D1 to D3 (Figure 2B). On the other hand, members of the genera *Lactobacillus* (D1: 0.1%, D2: 0.3%, D3: 2.3%), *Defluviitoga* (D1: 0.3%, D2: 1.1%, D3: 1.5%), *Limosilactobacillus* (D1: 0.1%, D2: 0.3%, D3: 1.3%), *Thermoclostridium* (D1: 0.6%, D2: 0.6%, D3: 1.2%) and *Methanothermobacter* (D1: 0.1%, D2: 0.1%, D3: 1.0%) showed an increasing relative abundance from D1 to D3, with partly much higher shares of these taxa in D3. Of the twelve most abundant genera, *Acetivibrio* (D1: 2.2%, D2: 2.7%, D3: 2.0%) was the only genus that showed a slightly higher share within digester 2 compared to digesters 1 and 3. Other genera, such as *Clostridium* (D1: 1.2%, D2: 1.1%, D3: 1.2%) and *Acetomicrobium* (D1: 0.7%, D2: 1.0%, D3: 1.0%), were more evenly distributed between the three digesters and seemed to be more resilient to different process conditions regarding temperature, fed substrates and ammonia concentrations than other genera (Figure 2B). At the genus level, about 58%, 60% and 65% of the input reads remained taxonomically unassigned for D1, D2 and D3, respectively.

Distinctive taxonomic profiles, as well as similarities, emerged when comparing the biogas microbiomes among the three digesters (D1–D3) of BP35. These differences were particularly evident at higher taxonomic levels, including superkingdom, phylum, order, and were even more pronounced at the genus level based on metagenomic single-read analyses.

**Figure 3:**
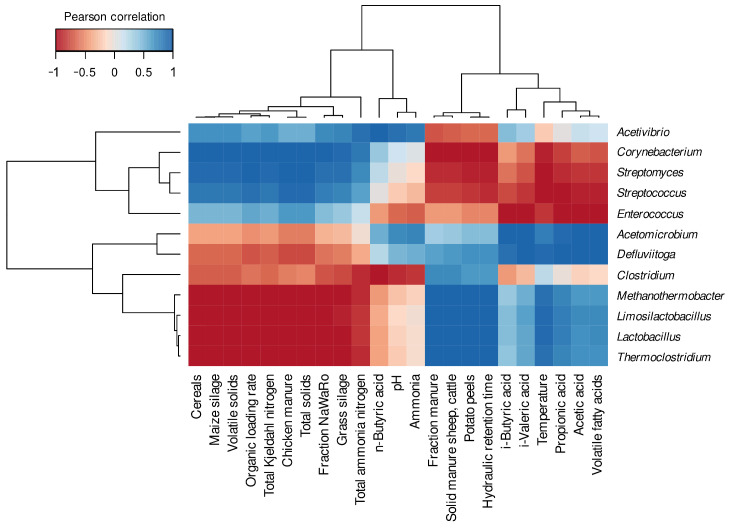
Clustered heatmap based on Pearson correlations of the twelve most abundant genera of biogas plant 35 based on metagenomic single-read analysis with the process and chemical analysis parameters.

To get an idea of potential parameters or conditions which may affect the abundance of certain genera in the three digesters, Pearson correlations were performed using the corresponding process and chemical analysis parameters (Figure 3), with the awareness that statistical analyses based on a limited number of samples can only provide insights into potential correlations.

It could be shown that the genus *Acetivibrio*, which had a slightly higher abundance in D2, showed a positive correlation with the concentration of *n*-Butyric acid, which exhibited the highest concentration in D2. Members of the genus *Acetivibrio* were previously observed in anaerobic digestion environments and described as cellulolytic organisms (e.g., [49]).

As expected, the genera that showed decreasing abundances from D1 to D3 (*Corynebacterium*, *Streptomyces*, *Streptococcus*, *Enterococcus*) showed correlations with the share of cereals, maize silage, chicken manure and the concentration of volatile solids (VS); these parameters were enhanced and more evenly distributed in D1 and D2 compared to D3. Furthermore, it was observed that the genera, which were abundant in digester 3 (*Defluviitoga*, *Methanothermobacter*, *Limosilactobacillus*, *Lactobacillus*, and *Thermoclostridium*), correlated positively with the fraction of manure, the amount of solid manure (sheep and cattle), the amount of potato peels, HRT and the process temperature. Members of the genera *Defluviitoga*, *Thermoclostridium* and the archaeal genus *Methanothermobacter* exhibited positive correlations with the process temperature, which is reasonable since members of these genera were described as thermophilic organisms [50,51,52]. On the other hand, the genera *Clostridium* and *Acetomicrobium*, which were more evenly distributed between the digesters, showed less positive or negative correlations with the process parameters than the other genera. However, one of the correlations of *Clostridium* is a negative correlation with the concentration of *n*-Butyric acid. The genus *Clostridium* is frequently observed within anaerobic digesters (e.g., [53,54,55,56]), and hydrolytic and fermentative members of this genus were described with a broad range of metabolic functions, such as cellulose and fiber degradation, and syntrophic acetate oxidation, as well as hydrogen generation (e.g., [57,58]. The genus *Acetomicrobium* showed several correlations with the concentrations of acids, volatile fatty acids (VFAs) and particularly acetic acid, which is in great accordance with the description of members of the genus *Acetomicrobiom* as acetate-producing organisms [59].

#### 3.2.2. Functional Potential (COG) of the Biogas Microbiomes Residing in the Three Digesters of Biogas Plant 35 Based on Single-Read Analyses

**Figure 4:**
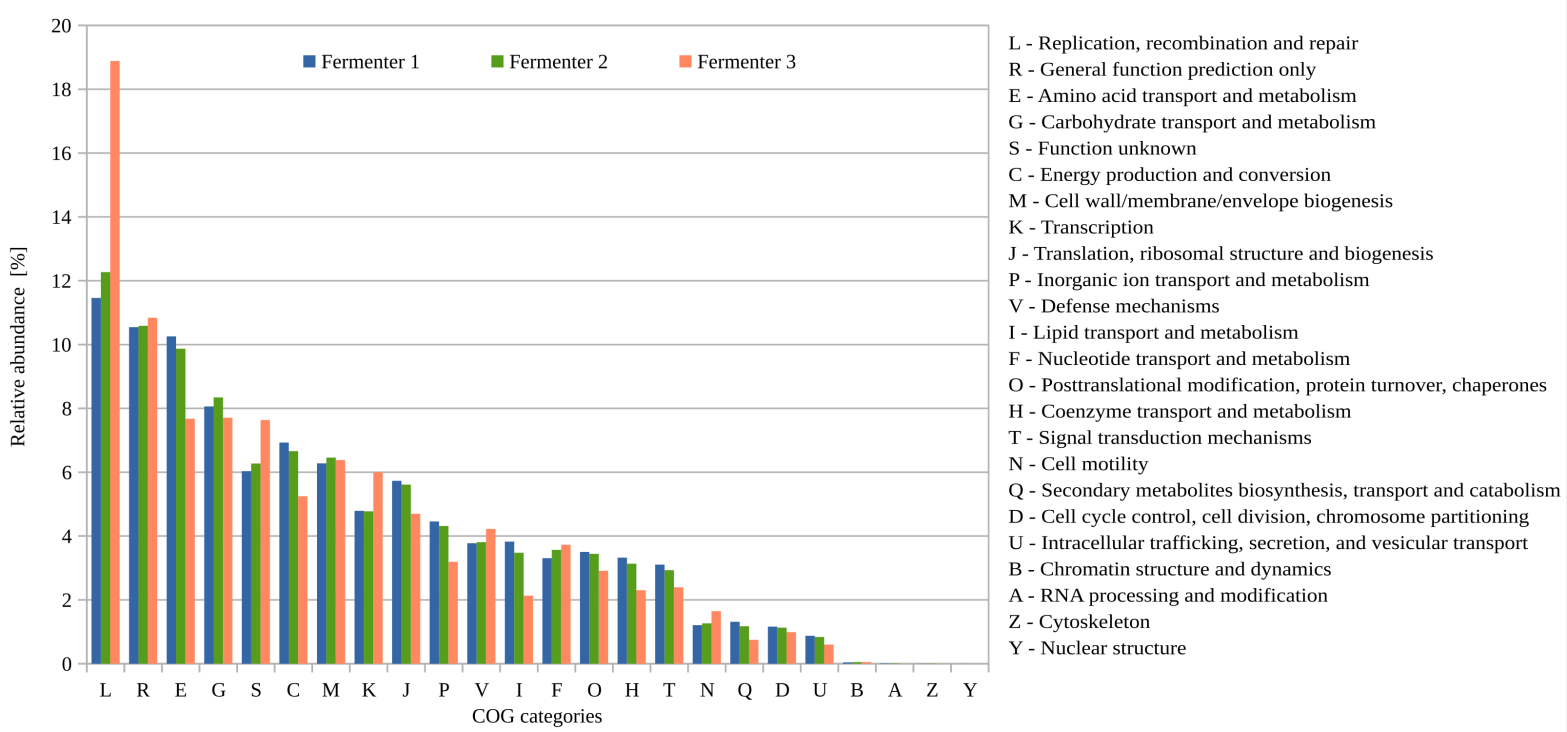
Relative abundance of cluster of orthologous groups of proteins (COG) categories for the microbiomes residing in the three digesters D1, D2 and D3 of biogas plant 35 based on normalized single-read analyses calculated within the metagenomics platform MGX [28].

Functional potential profiling of the BP35 microbiomes on a single-read basis according to cluster of orthologous groups of proteins (COG) revealed that the COG-categories L (replication, recombination and repair), E (amino acid transport and metabolism), G (carbohydrate transport and metabolism) and C (energy production and conversion) were among the most prominent ones (Figure 4).

This finding confirms previous COG-classifications for other full-scale biogas plants [60] and hence indicates the importance of these categories for biogas microbiomes. The only category that was clearly enriched in digester D3 (56.3 °C) is “replication, recombination and repair” (L). An explanation for this observation could be that under thermophilic conditions, recombination events and changes in DNA molecules happen more frequently. Therefore, microorganisms adapted to higher temperatures have to cope with rearrangements and alterations (probably also damage) of their genetic material by encoding appropriate repair systems. Moreover, external stressors such as elevated temperatures induce the lytic cycle of prophages integrated into the chromosomes of their hosts [61,62]. Accordingly, on average, microorganisms are more frequently infected by phages under thermophilic conditions compared to mesophilic systems. Interestingly, the COG number 4626 (Phage terminase-like protein) is enriched in digester D3. Phage terminases are involved in the packaging of DNA into phage proheads by the processing of DNA-concatemers [63,64,65].

### 3.3. Biogas Process-Related Functional Potential and Expressed Functions Resulting from Metagenome and Metaproteome Analyses Indicate Microbial Community Adaptations to the Process Conditions in the Three Digesters

The metagenomic paired-end reads were assembled yielding 447,603 contigs with a total length of 1,189,669,743 bp and an N50 value of 73,067 bp. Metagenomic sequencing reads were mapped back to the metagenome assembly with mapping rates of 82% for D1, 85% for D2 and 89% for D3. After gene prediction, coding sequences on all contigs were annotated using the KEGG database, resulting in functional KEGG orthology (KO) annotations for 1,158,091 of the 1,451,255 predicted genes. These KO assignments were used to calculate average genomic copy numbers (AGCNs, Section 2.3). Moreover, proteome spectral counts were assigned to genes and summed up on the level of KOs. The ACGNs and proteomic spectral counts were used to analyze the biogas process-related functional potential and expressed functions within the three digesters.

**Figure 5:**
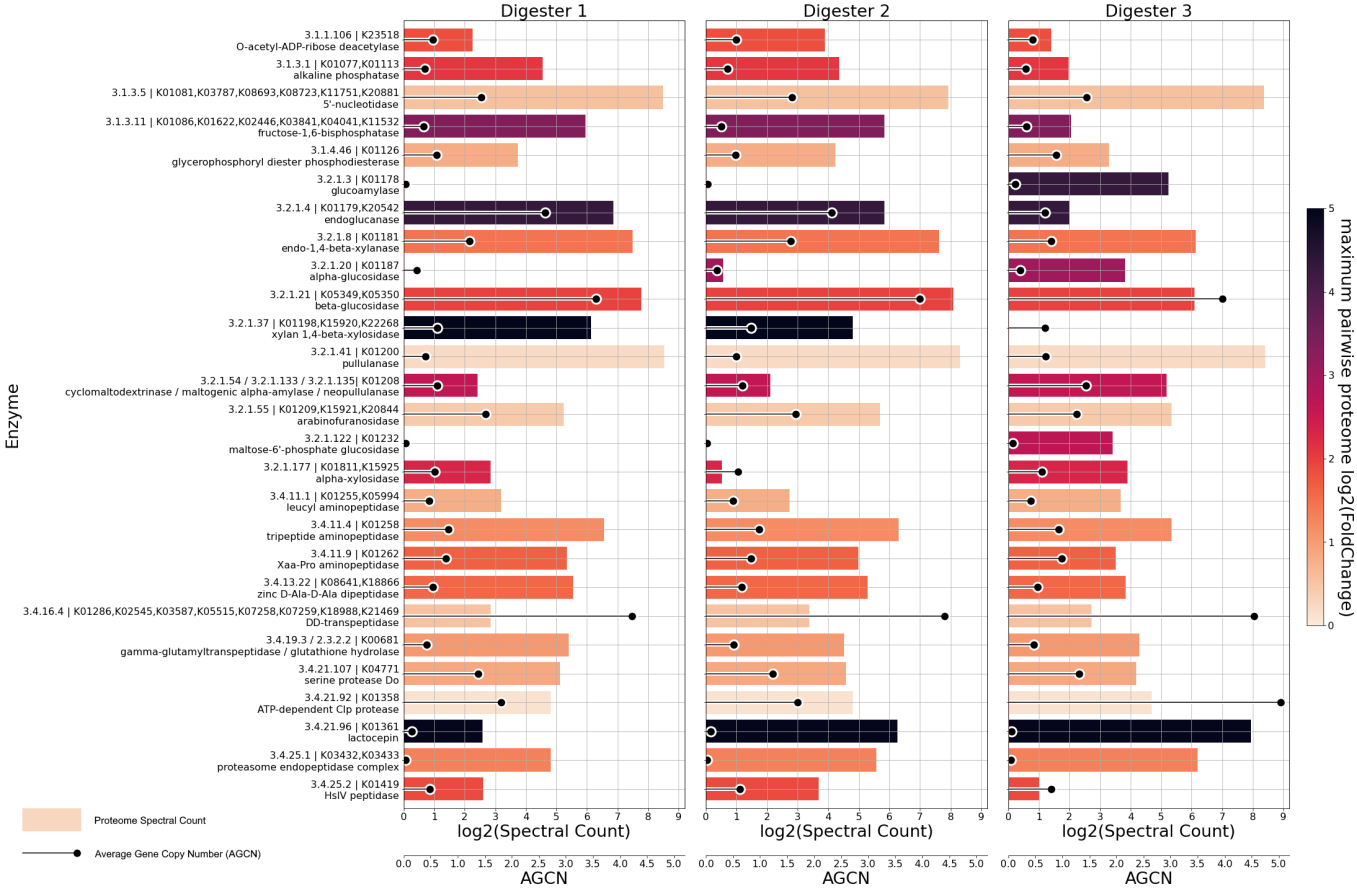
Average genomic copy numbers (black circles) and log-scaled proteome spectral counts (colored bars) of esterases (EC 3.1), glucosidases (EC 3.2.1) and peptidases (EC 3.4). Metrics for enzymes are calculated based on the result for related KEGG orthologies. Only enzymes exhibiting spectral counts ≥10 in at least one digester are shown. Colors of bars represent the maximum proteomic fold change that occurs between any of the three possible reactor pairings (D1/D2, D1/D3, D2/D3).

When comparing the genetic potential and metaproteome of the three microbial communities, the focus was laid on a subgroup of enzymes that are important for the biogas process. For this purpose, genes and enzymes involved in the hydrolysis for the breakdown of esters, peptides, complex carbohydrates and sugars were investigated. Corresponding metagenomic and metaproteomic abundances of esterases (EC 3.1), glucosidases (EC 3.2.1) and peptidases (EC 3.4) are shown in Figure 5. Furthermore, to analyze certain genes and enzymes involved in acidogenesis, acetogenesis and methanogenesis, the respective lists of key enzymes compiled by Sikora et al. [43] were considered.

Five esterases were detected with proteomic abundances above the threshold of 10 spectral counts. Proteomic abundances for alkaline phosphatase (EC 3.1.3.1) were similar in D1 and D2 but lower in D3. Enrichment of the enzyme was previously shown to be associated with an increase in the feed rate [66,67], which is lowest in D3 (Table 1).

Digesters 1 and 2 were fed with high shares of maize silage, which contains around 25 to 35% starch in dry matter [68] and cereals, while D3 was fed with potato peels containing about 52% starch in the dry mass [69]. Thus, it was expected to find enzymes required for starch utilization in all digesters. Glucoamylase (EC 3.2.1.3), alpha-glucosidase (EC 3.2.1.20) and maltose-6${}^{\prime }$-phosphate glucosidase (EC 3.2.1.122) are able to act in starch degradation processes; they were enriched in the proteome of digester 3. EC 3.2.1.41 comprises pullulanase types I and II, which are able to hydrolyze linkages in branched and linear polysaccharides and aid in the digestion of starch, for example by degrading amylopectin to smaller sugars [70]. These pullulanase enzymes showed even proteomic distribution across the different digesters.

A range of enzymes involved in the degradation of cellulose, xylose and other hemicelluloses were detected in all digesters. Proteomic abundance of endoglucanase (EC 3.2.1.4), endo-1,4-beta-xylanase (EC 3.2.1.8), beta-glucosidase (EC 3.2.1.21) and xylan 1-4-beta-xylosidase (EC 3.2.1.37) was lowest in digester 3. Genomic abundance only differed for endoglucanase, for which the lowest value was detected in D3. The substrate of digester 3 contained nearly 60% solid manure, while the substrate mix of digesters 1 and 2 was composed of less solid manure and more silage (see Table 1). While cattle manure contains significant amounts of cellulose (13–30%) and hemicellulose (14–32%) [71,72,73,74,75], it can be more recalcitrant than silage since easily accessible fiber is already degraded by enzymes in the digestive tract of cattle [76,77]. This might lead to a reduced need for cellulolytic enzymes in D3.

Differences in the proteomic abundance of peptidases were less pronounced than those of glucosidases, with the exceptions of lactocepin (EC 3.4.21.96), an endopeptidase usually found in lactic acid *Bacteria* [78], and HslV peptidase (EC 3.4.25.2). The latter is part of an intracellular proteasome-like complex, which, in *Escherichia coli*, degrades damaged proteins as part of the heat shock response [79,80]. It was most abundant in the proteome of D2 and least abundant in the proteome of D3, though genomic abundance was highest in D3.

**Figure 6:**
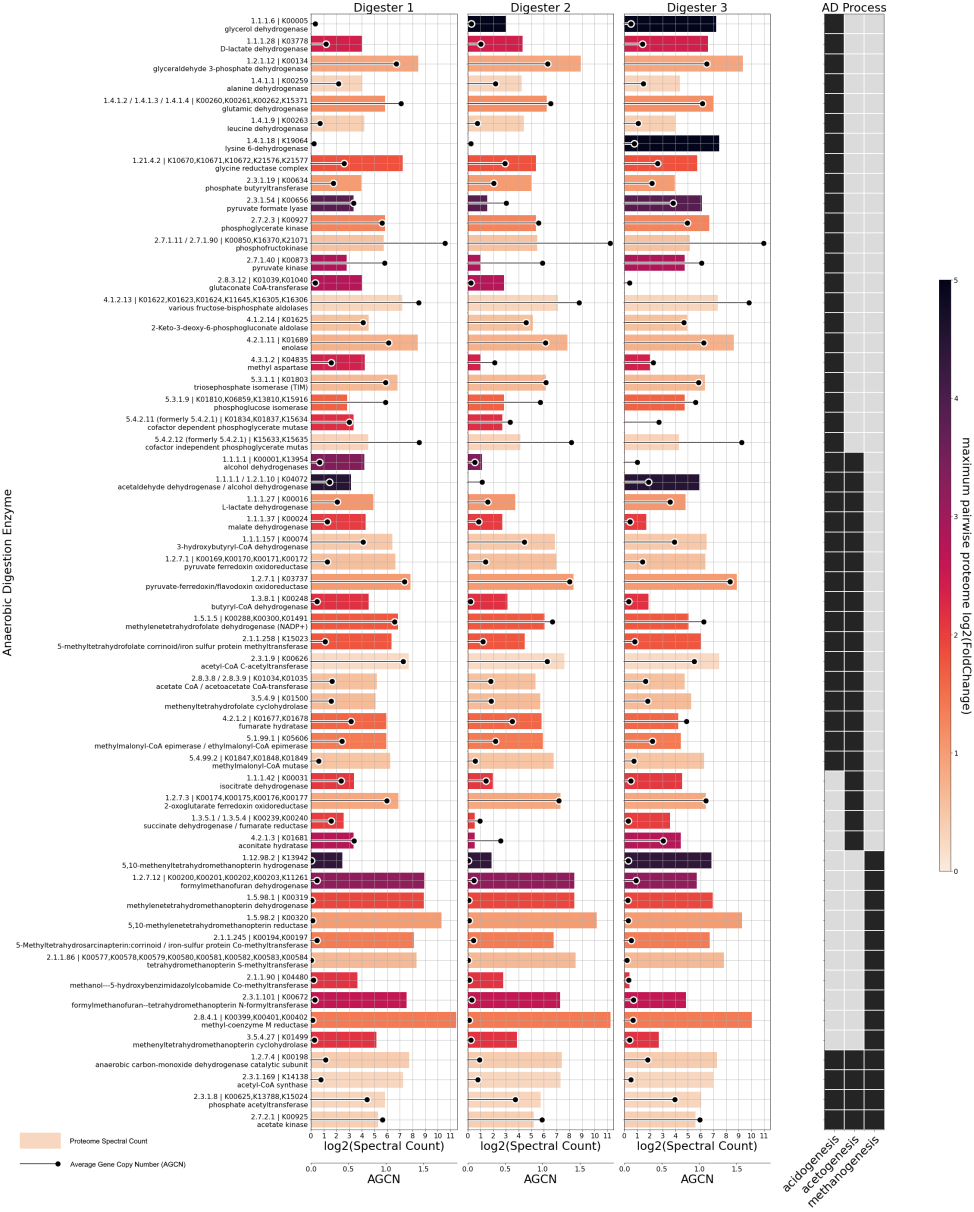
Average genomic copy numbers (black circles) and log-scaled proteome spectral counts (colored bars) of anaerobic digestion (AD) key enzymes in acido-, aceto- and methanogenesis [43] in the three digesters. KEGG orthology (KO) data were manually summarized at enzyme level by summing up AGCNs of KOs with similar functionality and taking the median AGCN where KOs represent individual subunits of an enzyme. Spectral counts were always summed up. Only entries exhibiting a spectral count of 10 or higher in at least one digester are shown. Colors of bars represent the maximum proteomic fold change that occurs between any of the three possible reactor pairings (D1 vs. D2, D1 vs. D3, and D2 vs. D3).

To further unravel the genomic potential and the expressed functions of the microbial communities in the three digesters, key enzymes involved in the acidogenesis, acetogenesis and methanogenesis steps of the anaerobic digestion process were selected [43]. Genomic and proteomic abundances across the three digesters are shown in Figure 6 for those enzymes, featuring spectral counts of at least 10 in at least one digester.

The analysis revealed a portion of genes and enzymes exhibiting very similar abundances across digesters as well as genes and gene products, where abundance is not uniform. Glycerol dehydrogenase (EC 1.1.1.6) exhibited the highest proteomic fold change between digesters, with high proteomic abundance in digester 3, low abundance in digester 2, and no protein spectral counts detected in digester 1. This is surprising since neither glycerol nor any substrates rich in lipids were part of the feed of the digesters. However, cow manure may contain more than 10% lipids as a fraction of the total solids [81]. D3 had the highest fraction of cow manure as the substrate (Table 1). Since some known glycerol dehydrogenases possess a broad substrate specificity, the detected enzymes may also be involved in other processes like 2,3-butanediol fermentation or the reduction of (di-)hydroxyacetone [82,83,84]. The Wood–Ljungdahl pathway is represented by six key enzymes detected at the proteome level in all digesters (ECs 1.2.7.4, 1.5.1.5, 2.1.1.258, 2.3.1.169, 3.5.4.9, 6.3.4.3; Figure 6). For each of these enzymes, most spectral counts were detected in D1, with methylenetetrahydrofolate dehydrogenase (EC 1.5.1.5) and 5-methyltetrahydrofolate:corrinoid/iron–sulfur protein Co-methyltransferase (EC 2.1.1.258) exhibiting the highest differences. With the exception of EC 2.1.1.258, these enzymes were least abundant in D3, though all Wood–Ljungdahl pathway enzymes were still well represented across all digesters.

Multiple enzymes (e.g., ECs 1.1.1.42, 1.3.5.1, and 4.2.1.3) involved in acetogenesis showed the same pattern of highest proteomic abundance in digester 3 and lowest in digester 2. These are essential for syntrophic oxidation of acetate as described by Galushko and Schink [85] and might indicate a preference for this type of acetate utilization coupled with hydrogenotrophic methanogenesis in the microbiome of D3.

Key glycolysis enzymes which were detected (ECs 5.3.1.9, 2.7.1.11, 4.1.2.13, 5.3.1.1, 1.2.1.12, 2.7.2.3, 5.4.2.11, 5.4.2.12, and 4.2.1.11) showed only slight differences in genomic abundance across digesters. The same was observed regarding proteomic abundance, with the exception of phosphoglucose isomerase (EC 5.3.1.9), which was enriched in the proteome of D3. Bifunctional acetaldehyde-CoA/alcohol dehydrogenase (EC 1.2.1.10 / 1.1.1.1) was most abundant at genome and proteome levels in D3, while no proteomic spectral counts were detected in D2. The presence of the enzyme might be related to microorganisms performing ethanol oxidation as described by Bertsch et al. [86]. Alcohol dehydrogenase (EC 1.1.1.1), providing another avenue for ethanol transformation, was not detected in D3 and was most abundant in D1. Malate dehydrogenase (EC 1.1.1.37), which may catalyze reactions in acidogenesis as well as acetogenesis, was most abundant in D1 and least abundant in D3 on the proteome as well as the genome level. On the proteome level, the same pattern can be observed for butyryl-CoA dehydrogenase (EC 1.3.8.1), which catalyzes the second step of butyrate oxidation [87,88].

When examining the proteomic abundance of enzymes involved in methanogenesis, hydrogen:5,10-methenyltetrahydromethanopterin oxidoreductase (EC 1.12.98.2) stands out as the only methanogenesis enzyme most abundant in digester 3. While it is a key enzyme in hydrogenotrophic methanogenesis, 5,10-methylenetetrahydromethanopterin:coenzyme-F420 oxidoreductase (EC 1.5.98.1) can fulfill a similar role and was more abundant in D1 and D2 compared to D3. All other key enzymes exclusively involved in hydrogenotrophic methanogenesis (ECs 1.2.7.12, 1.5.98.2, 2.3.1.101, 3.5.4.27) were detected in all digesters and were most abundant in digester 1, while exhibiting the lowest abundance in digester 3. The only detected enzyme exclusively involved in methylotrophic methanogenesis (5-methyltetrahydrosarcinapterin-corrinoid/iron-sulfur protein Co-methyltransferase, EC 2.1.1.245) showed a similar pattern with the highest spectral counts in D1 and lowest in D3. Other enzymes characteristic for the methylotrophic pathway (ECs 2.1.1.246, 2.1.1.247, 2.1.1.248, 2.1.1.249) were not detected at the proteome level and were thus excluded from Figure 6. The enzyme Co-methyltransferase (EC 2.1.1.245), important in the acetotrophic methanogenesis pathway, was also most abundant in digester 1 and least abundant in digester 3. Overall, the fraction of the proteome dedicated to methanogenesis was highest in digester 1 and lowest in digester 3. Upon investigating the genomic abundance of methanogenesis genes (Figure 6), an opposing trend was observed since all ten methanogenesis genes showed the highest abundance in digester 3. This indicates a lower activity of the individual methanogenic archaeal cells in D3, possibly related to the available substrates. As mentioned before, the feed of digester 3 contained almost 60% cow manure, which is more resistant to anaerobic digestion compared to the silage and cereals that were fed to D1 and D2 [89]. Furthermore, digester 3 had the highest HRT and lowest OLR among the three digesters investigated in this study.

For a subset of the enzymes shown in Figure 5 and Figure 6, differences in the metagenomic abundance of a gene correlate with differences in the proteomic abundance. Examples are glucoamylase (EC 3.2.1.3), endoglucanase (EC 3.2.1.4), lysine 6-dehydrogenase (EC 1.4.1.18) and 5,10-methenyltetrahydromethanopterin hydrogenase (EC 1.12.98.2). However, most differentially abundant enzymes do not exhibit a corresponding difference in the abundance of related genes, and in several cases, the two metrics even show opposing trends, such as with lactocepin (EC 3.4.21.96), alcohol dehydrogenase (EC 1.1.1.1), isocitrate dehydrogenase (EC 1.1.1.42) and formylmethanofuran dehydrogenase (EC 1.2.7.12). These observations demonstrate that, as expected, metagenomic abundances of genes are not a reliable indicator of the actually perfomed metabolic functions or of differences in the metabolic activity of microbial communities.

Overall, the combined analysis of the biogas process-related functional potential and the expressed functions of the whole microbial communities within the three different biogas digesters revealed that the relevant anaerobic digestion key genes and their encoded enzymes were present and also expressed. As hypothesized, the results revealed a much broader and more diverse functional potential of the microbial communities based on the metagenome compared to the actual expressed functions based on the metaproteome. Differences between the metagenomic and metaproteomic abundances of certain genes and enzymes, respectively, could mostly be explained by higher or lower gene expression of the microbial communities in response to differences in process parameters between the digesters. However, methodological limitations of the metaproteomics approach may also have an impact on the observed differences.

### 3.4. MAG-Centric Metagenome and Metaproteome Analyses Enabled Characterization of the Functional Potential, Expressed Functions and Role of Specific MAGs in the Biogas Process

To determine the metagenome-based relative abundances of MAGs in the three main digesters of BP35, reads from all nine metagenomic samples were mapped back to all assembled contigs. Relative abundances were calculated for each MAG, also taking into account the fraction of reads mapping to unbinned contigs in order to determine the MAG abundances in relation to the whole microbial community. It appeared that individual replicates from each digester feature very similar MAG abundance profiles, supporting the reliability of the analysis (Supplement Table S2). Hence, replicates were combined to compute median abundances values for each MAG in each digester. The metaproteome-based relative abundances were calculated based on all proteins, which were assigned uniquely to the metagenomic contigs. The proteomic spectral counts were normalized based on the total spectral counts for each digester and finally by using the size and completeness of the corresponding MAG (Section 2.5). Since only high-quality MAGs should be considered for further analyses, all MAGs above 50% completeness, below 10% contamination and above 0.5% relative abundance either in the metagenomes or the metaproteomes of the three digesters were considered (Table 2). After stringent filtering, 46 (44 bacterial, 2 archaeal) of a total of 300 (294 bacterial, 6 archaeal) MAGs remained as high-quality MAGs, and about 46.21% up to 56.50% of all mapped reads from the three digesters were attributed solely to these 46 MAGs. The MAG-centric metaproteome analysis revealed that a total of 45.02%, 48.42% and 48.02% of the unique metaproteins were solely assigned to the 46 high quality MAGs of the digesters 1, 2 and 3 (Table 2).

**Figure 7:**
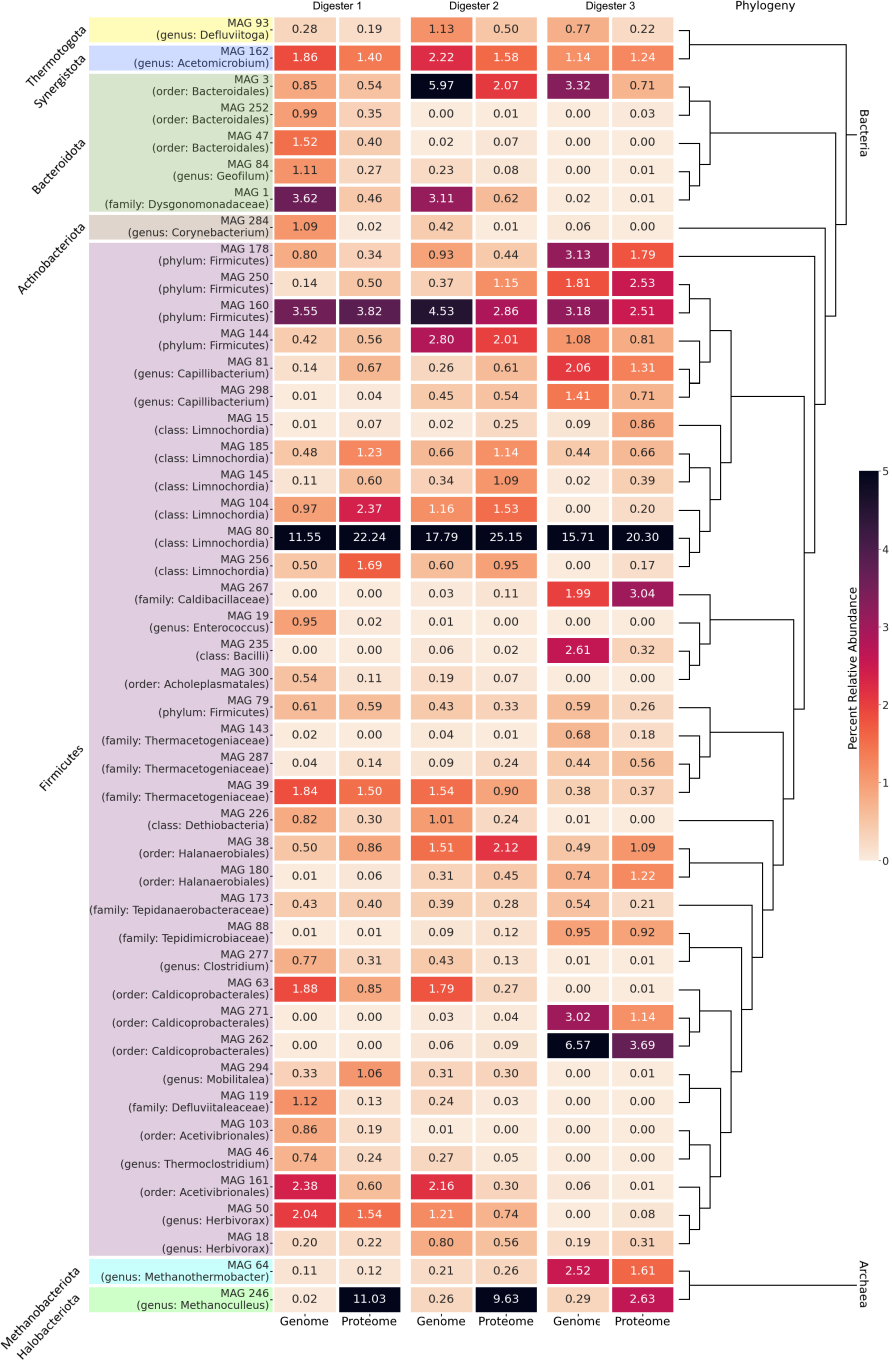
Relative genomic and proteomic abundances of the 46 high-quality MAGs in the three digesters of BP35 calculated based on mapped reads and assigned metaproteins. Taxonomic assignment of the MAGs is based on the GTDB taxonomy. Placement in the phylogenetic tree is based on GTDB-tk output. Branch lengths do not represent phylogenetic distance.

The MAG-centric metagenome and metaproteome abundance profiles were plotted as heatmaps for the three digesters (Figure 7). The 46 high-quality MAGs showed metagenome-based relative abundances from close to 0 up to 17.8% in the three digesters, and the MAG-centric metaprotein-based approach revealed relative abundances from 0.02% to 25.13% for the 46 different MAGs (Figure 7).

It became obvious that the MAG relative abundances based on the metagenome, and the actual expressed functions, as revealed by the relative abundances of the assigned metaproteins, mostly show comparable values within the same digester. However, in some cases, these values differ strongly for certain MAGs. This indicates that the genomic abundance of organisms and their protein expression activity in biogas digesters do not always correlate. Similar observations were reported previously for *Bacteria* and especially for methanogenic *Archaea* that seem to be disproportionately active in biogas digesters [90,91,92]. In this study, MAG 246 exhibits very high proteomic abundances, while showing less than 1% relative abundance in the metagenome. It was previously observed in biogas metaproteome studies that a high percentage of spectral counts was assigned to methanogens, especially to their methanogenesis enzymes, while the relative genomic abundance of those taxa in the community tends to be much lower [19,93,94]. While this might explain observations in the digesters D2 and D3, highest proteomic abundance and lowest genomic abundance for MAG 246 was observed in digester 1. The reason might be that related methanogens are present in the microbial community but their methanogenesis genes were not assembled, leading to spectral counts being potentially falsely attributed to a certain MAG. However, this reason could be excluded for MAG 246 since no evidence for another archaeal and closely related MAG or species was found.

In summary, MAGs with different relative proteomic/genomic abundances in the three digesters were identified based on log2 fold changes. Here, especially the relative MAG abundances of digester 3 compared to digester 1 and 2 showed the strongest differences, which is in accordance with the fact that D3 also differed strongly from D1 and D2 regarding the process parameters. However, also MAGs with more even distributions in the three digesters were identified, indicating resilience of the corresponding species to different process conditions within these digesters.

In order to analyze the potential role of certain MAGs in the biogas process, the genomic potential and proteomic expression of biogas process-related key enzymes and pathways, as described by Sikora et al. [43], were investigated for ten differentially abundant MAGs and four evenly distributed MAGs in the three digesters.

#### 3.4.1. Differentially Abundant High-Quality MAGs Showed Adaptations to the Different Process Conditions and Their Role in the Three Digesters Was Deduced Based on MAG-Centric Metaproteomics

**Figure 8:**
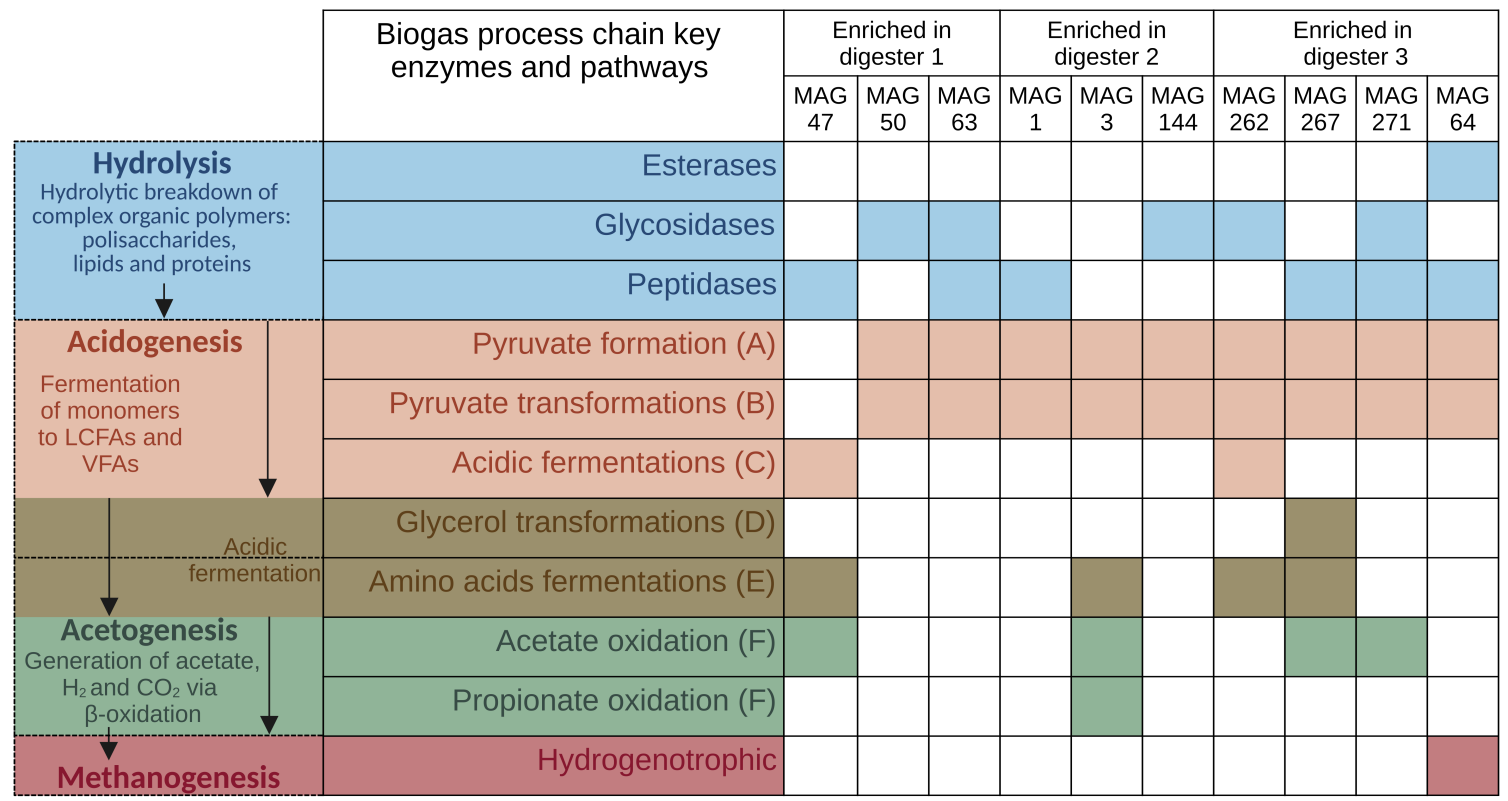
Proteomic expression of biogas process chain key enzymes and pathways for ten differentially abundant MAGs in the three biogas digesters. Key enzymes and pathways, as well as the categorization (A–F), were derived from Sikora et al. [43] and were marked when one key enzyme was present based on the metaproteome counts.

While the genetic potential of MAGs is often described in the literature, their actually expressed metabolic functions in differently operated digesters remain mostly unknown. Thus, MAG-centric metagenome- and metaproteome-based relative abundances were used to identify ten differentially abundant and differentially active MAGs in the digesters D1, D2 and D3 for in-depth analysis. Moreover, this approach enabled the detailed characterization of MAGs based on their biogas process-related functions and key enzymes (Figure 8) as well as highly expressed enzymes and KEGG modules (Table S3) in order to determine their role in the biogas process chain. This represents a special opportunity that has not yet been sufficiently exploited in the field of biogas microbiome research.

##### MAGs Most Abundant and Active in Digester 1 (D1)

Several MAGs showed the highest genomic and proteomic abundance in D1. The most predominant were MAG 50 and MAG 63, which also showed high abundance in D2, and MAG 47, which was present in D2 with only 0.02% relative genomic abundance and was not detected in D3.

MAG 47 was classified as a member of the order *Bacteroidales* (GTDB: Bact-19 sp002412425; NCBI: GCA_002412425.1) with a > 99.6% average nucleotide identity (ANI) to the closest relative MAG GCA_002412425.1 [6]. Protein spectra for 30 of the functionally annotated genes of MAG 47 were detected in the samples from D1. The genome of this MAG encodes 51 peptidases (EC 3.4) and a peptidyl dipeptidase (K01284), which was detected at the proteome level in D1. Furthermore, MAG 47 has the genomic potential for the degradation pathways of alanine, serine, glycine, glutamate, glutamine, histidine, aspartate, asparagine, lysine, methionine, valine, (iso)leucine and tryptophane. Several enzymes of these pathways (K00626, K00262, K00600, K01077, K18013) and two submodules of 2-ketoglutarate ferredoxin oxidoreductase (K00175, K001773) are among the 30 orthologies detected on the proteome level in D1. Also detected in the MAG 47 proteome in D1 were enolase (K01689) and acetate-CoA/acetoacetate-CoA-transferase (K01034, K01035), along with the genomic potential for the transformation of Acetyl-CoA to butanoate. When summing up spectral counts of subunits encoded by the *hndABCD* operon (K18330, K17992, K18331, K18332), the Hnd hydrogenase complex is the enzyme with the second-highest spectral counts. While only hydrogen-oxidizing activity is reported for the enzyme of *Desulfovibrio fructosovorans* [95,96,97,98], similar enzymes in other organisms might catalyze hydrogen evolution [99,100,101]. The results indicate that MAG 47 acts as a proteolytic amino acid degrader in the community of D1 (Figure 8), which is in line with previous findings regarding members of the *Bacteroidales* order [102].

MAG 50 was detected with a relative genomic abundance of 2.04%, 1.21% and below 0.01% in digesters one, two and three, respectively. Relative proteomic abundance was 1.54% in D1 but only 0.74% in D2, indicating a higher activity per cell in D1. This MAG was classified as a member of the genus *Herbivorax* (GTDB: *Herbivorax* sp012517995; NCBI: GCA_012517995.1) based on an ANI greater than 99.7% to the closest related MAG, which was binned before by Parks et al. [6]. The genus was previously described by Koeck et al. [103] and is synonymous with the genus *Acetivibrio* [104], whose abundance was shown to correlate positively to the concentration of *n*-butyric acid (Section 3.2.1, Figure 3). The strain type *Herbivorax saccincola* GGR1${}^{T}$ sequenced by Koeck et al. [103] was isolated from a biogas digester fed with cow manure and maize silage and operated at 55 °C. The strain GGR1${}^{T}$ is able to utilize cellulose, galactose, xylan, xylose, glucose and sorbitol, and produces hydrogen, ethanol and acetate during fermentation. Likewise, MAG 50 has the genomic potential for (hemi-)cellulose degradation, and the specific proteome of the MAG detected in D1 and D2 revealed the presence of related enzymes: the genome encoded 21 endoglucanase genes (K01179), with related gene products for three of these detected in the proteome in D1 as well as D2. A xylose-isomerase (K01805) and endo-1,4-xylanase (K01181) were also detected at the proteome level in digesters 1 and 2. Genes encoding beta-glucosidase (K05349), endo-beta-1,4-mannase (K01218), alpha-glucoronidase (K01235) and beta-mannosidase (K01192) were present in the MAG 50 assembly. Furthermore, the genome encoded all nine subunits of V/A-type H${}^{+}$/Na${}^{+}$-transporting ATPase with four subunits detected in the proteome of D1 and two in D2. Similar to MAG 47, genes for a NADP-reducing hydrogenase (*hndABCD*, K18330, K17992, K18331, and K18332) were found in MAG 50 with one subunit detected in the proteome of D1. The genome comprised the complete KEGG glycolysis module (M00001). Five glycolysis enzymes were found in the proteome of D1 and D2, and four others were detected only in the D1 proteome. Genome and proteome data indicate that the primary role of MAG 50 is the breakdown of polysaccharides, such as cellulose and hemicellulose, and the generation of molecular hydrogen as commonly observed in *Herbivorax* species (e.g., [103]).

MAG 63 exhibits similar relative genomic abundances in digesters 1 (1.88%) and 2 (1.79%) and less than 0.01% in digester 3. Relative proteomic abundance, however, is higher in D1 (0.85%) compared to D2 (0.27%), indicating the higher metabolic activity of this organism in D1. The MAG was classified as belonging to the class *Clostridia* and assigned to the order *Caldicoprobacterales* (GTDB: UBA3906 sp002391555; NCBI: GCA_002391555.1) with an ANI greater than 99.3% to the corresponding MAG [6]. *Caldicoprobacterales* are part of the complex microbial communities found in anaerobic digestion processes, such as those occurring in biogas reactors [105,106]. Some members of this order were described to possess cellulolytic capabilities, allowing them to participate in the degradation of complex organic materials, including cellulose, hemicellulose, and lignocellulosic biomass [107]. While the first carbon oxidation module of the citrate cycle (M00010) is complete, the second (M00011) is not. Four enzymes involved in glycolysis and pyruvate metabolism were found in the MAG 63 proteome of D1 and D2 with four more only detected in the D1 proteome. Three proteins involved in (hemi-)cellulose degradation were found in the proteome. Xylose isomerase (K01805) was present in both digesters, while cellobiose phosphorylase (K00702) and xylulokinase (K00854) were only detected in D1. Genes encoding endoglucanase (K01179), endo-1,4-xylanase (K01181), beta-glucosidase (K05349) and oligosaccharide reducing-end xylanase (K15531) are encoded on MAG 63. ABC.MS.S (K02027) transporters facilitating carbohydrate uptake have the most spectral counts assigned to them in D1 as well as D2. Another transporter found in the organism’s proteome in both reactors is adenine/guanine/hypoxanthine permease (K06901), while branched-chain amino acid transport system substrate-binding protein (K01999), putative tryptophan/tyrosine transport system substrate-binding protein (K010989) and D-methionine transport system substrate-binding protein (K02073) were present in the D1 proteome. The latter, along with the presence of s-adenosylmethionine synthetase (K00789) and adenosylhomocysteinase (K01251) in the proteome of D1, might be an indication of methionine degradation being carried out by the organism. Similar to the MAGs discussed before, MAG 63 encodes a NADP-reducing hydrogenase (*hndABC*, K18330, K17992, K18331), and the presence of the corresponding proteins can be detected in both digesters. Investigation of the genome and proteome suggests that MAG 63 is able to degrade cellulose and hemicelluloses, imports amino acids from the environment and produces hydrogen. The metagenome–metaproteome ratio indicates that individual MAG cells are more active in digester 1 compared to digester 2.

##### MAGs Most Abundant and Active in Digester 2 (D2)

MAG 1 is enriched in digester 1 and 2 compared to digester 3 (metagenome: D1 3.62%, D2 3.11%, D3 0.02%). Based on the metaproteome, this MAG showed the highest activity in D2 with 0.62% (D1 0.46%, D3 0.01%). This MAG was taxonomically classified as a member of the family *Dysgonomonadaceae* (GTDB: UBA4179 sp002381125; NCBI: GCA_002381125.1). The closest relative is a MAG, which was assembled before by Parks et al. [6] with an ANI of 98.9%. Members of the family *Dysgonomonadaceae* were frequently found to be abundant in biogas digesters with increased ammonium concentrations (e.g., [14,108]). Interestingly, higher ammonia concentrations were also observed within digester 2 of this biogas plant (Table S1). This aligns with prior findings, as it was previously hypothesized that members of this family exhibit a notable capacity to effectively adapt to stressful and/or challenging environmental conditions [14]. Based on the relative abundance of the proteomic spectral counts, KEGG modules that are related to the biogas pathway were abundant for this MAG, such as the modules for glycolysis (M00002, 5.8% D2), glycogen degradation (M00855, 0.9% D2) as well as pyruvate oxidation (M00307, 0.6% D2). Within this MAG, genes which belong to the hydrolysis were among the highest expressed genes, such as a serine protease (EC 3.4.21.107), an ATP-dependent Clp protease (EC 3.4.21.92) and a xylose isomerase (K01805). Proteases are involved in the degradation of proteinaceous compounds, whereas the ATP-dependent Clp protease is more likely linked to stress response and regulatory functions [109], and the xylose isomerase is involved in the breakdown of carbohydrates. Moreover, a potential polysaccharide utilization locus (PUL) composed of the two SusC- and SusD-like proteins is highly expressed by MAG 1. PULs are a unique feature of the phylum *Bacteroidetes* to sense nutrients and enable glycan digestion [110,111], which underlines the hydrolytic role of MAG 1. Acidogenesis key enzymes were also identified for this MAG, which were mainly expressed in D2. These comprise a highly expressed enolase (EC 4.2.1.11) which is involved in the Emden–Meyerhof pathway of the glycolysis and a phosphate acetyltransferase (2.3.1.8) which is involved in glycolytic fermentations to acetate. Moreover, a succinate dehydrogenase/fumarate reductase (EC 1.3.5.1), a methylmalonyl-CoA/ethylmalonyl-CoA epimerase (EC 5.1.99.1) and a glutaconyl-CoA/methylmalonyl-CoA decarboxylase were expressed by this MAG. These enzymes indicate propionate generation via parts of the citrate cycle and the methylmalonyl-CoA pathway, which was also hypothesized for the *Dysgonomonadaceae* isolate ING2-E5B by Hahnke et al. [112]. Thus, MAG 1 can be described as a proteolytic and hydrolytic acidogenic member of the family *Dysgonomonadaceae* that produces acetate and propionate.

MAGs 3 and 144 are enriched in digester 2 with relative abundances of 5.97% and 2.80% based on the metagenome and 2.07% and 2.01% based on the metaproteome. In digester 1 and 3, the metagenome-based abundances were lower for MAG 3 (D1 0.85%, D3 3.32%) and MAG 144 (D1 0.42%, D3 1.08%) as well as the abundances based on the metaproteome of MAG 3 (D1 0.54%, D3 0.71%) and MAG 144 (D1 0.56%, D3 0.81%).

MAG 3 was assigned as a member of the order *Bacteroidales* (GTDB: DTU049 sp001512885; NCBI: GCA_001512885.1) with an ANI of 99.9% to a closely related MAG that was assembled before by Campanaro et al. [113] and taxonomically classified as *Rikenellaceae* sp. DTU001. The KEGG modules for serine biosynthesis (M00020), glycolysis (M00001) and gluconeogenesis (M00003) were most abundant based on the metaproteome. The biogas-related genes and enzymes that showed the highest expression were a fructose-bisphosphate aldolase (EC 4.1.2.13) and a triosephosphate isomerase (EC 5.3.1.1) belonging to the glycolysis via the Embden–Meyerhof pathway; a glutamate dehydrogenase (NADP${}^{+}$) involved in acidic fermentations; and 2-oxoglutarate/2-oxoacid ferredoxin oxidoreductase, which is a key enzyme during acetate oxidation. This MAG also harbors the hydrogenase complex HndABCD as described above for MAG 47, 50 and 63. Like MAG 1, MAG 3 showed expression of methylmalonyl-CoA mutase, methylmalonyl-CoA/ethylmalonyl-CoA epimerase, and glutaconyl-CoA/methylmalonyl-CoA decarboxylase, which might indicate propionate generation or syntrophic propionate oxidation via the methylmalonyl-CoA pathway. Syntrophic propionate oxidation seems reasonable for this MAG since it encodes several functional domains involved in electron transfer, such as NiFe hydrogenases, RnfABCDEG, EtfAB and cytochrome c as well as syntrophy-specific domains, like FtsW, RodA and SpoVe [43]. Based on the expression pattern, this MAG can be described as an acido- and acetogenic member of the *Bacteroidales*, which might oxidize propionate.

MAG 144 belongs to the phylum *Firmicutes*_G (GTDB: JAAYGG01 sp012727895; NCBI: GCA_012727895.1) with an ANI of 99.7% to the closest related MAG, which was metagenomically assembled before from an anaerobic digester metagenome by Campanaro et al. [8]. The most abundant KEGG modules of this MAG were the glycolysis (M00001), gluconeogenesis (M00003), the semi-phosphorylative Entner–Doudoroff (M00308), and the pyruvate oxidation (M00307) pathways. The highest expressed genes and enzymes belong to aldouronate (*lplA*, K17318) and raffinose/stachyose/melibiose (*msmE*, K10117) transport systems, as well as to several hydrolytic enzymes, like pullulanase, endo-1,4-beta-xylanase, and the ATP-dependent Clp protease. As already described for MAG 1, the ATP-dependent Clp protease could be linked to stress response and regulatory functions. Moreover, this MAG expressed glycolytic enzymes like glyceraldehyde 3-phosphate dehydrogenase, phosphoglycerate kinase, 6-phosphofructokinase, triosephosphate isomerase and xylose isomerase, as well as enzymes involved in glycolytic fermentations, like pyruvate ferredoxin oxidoreductase and phosphate acetyltransferase. Members of the phylum *Firmicutes* are known to be ubiquitous in microbiomes of biogas digesters (e.g., [4,56,114]) and to harbor a wide range of enzymes involved in the degradation of complex polysaccharides like hemicellulose, cellulose and xylan (e.g., [115]). Investigation of the MAG 144 genome and proteome suggests that this organism is a hydrolytic and acidogenic member of the *Firmicutes*, with the potential to degrade starch and xylose.

##### MAGs Most Abundant and Active in Digester 3 (D3)

It became obvious that the MAG abundance patterns of digesters 1 and 2 are more similar to each other as compared to digester 3, whose pattern is most different (Figure 7). The bacterial MAGs 267, 262, 271 and the archaeal MAG 64 were the most enriched and active in D3 compared to D1 and D2. It appeared that these MAGs adapted to deal with the distinct feed mix (Table 1) and the high process temperature in D3 (56.3 °C) compared to D1 (44.5 °C) and D2 (50.0 °C).

MAG 262 and MAG 271 are members of the order *Caldicoprobacterales* of the class *Clostridia*, whereby these MAGs could not be assigned to a GTDB species cluster. For both MAGs, the closest relative in GTDB is the MAG JAAYGY01 sp012522165 (NCBI: GCA_ 012522165.1), which was reconstructed from an anaerobic digester metagenome [6], with an ANI of 86.1% for MAG 262 and 86.6% for MAG 271. Thus, the two MAGs are most likely new species and may even represent a new genus. The prefix *caldi* (from Latin “*caldus*”: warm, hot) in the order name of these MAGs already indicates that corresponding species are possibly adapted to higher temperatures. The taxon *Caldicoprobacterales* was introduced in GTDB as a new group at the taxonomic order level. *Caldicoprobacter oshimai* DSM21659T is a GTDB species representative of the *Caldicoprobacterales* and also represents a NCBI-type strain [107]. *C. oshimai* was isolated from sheep feces and features a thermophilic and xylanolytic lifestyle. In accordance with this, digester 3 received high amounts of sheep and cattle manure (59.3%) in contrast to D1 and D2 (Table 1). Analysis of MAG 262 and MAG 271 revealed that they both possess complete or almost complete KEGG modules for pyruvate oxidation (M00307), the citrate cycle (M00010), the pentose phosphate pathway (M00007), galacturonate degradation (M00631), glucuronate degradation (M00061) and galactose degradation (M00632). MAG 262 also features the phosphate acetyltransferase/acetate kinase pathway for ATP generation yielding acetate. MAG 262 showed the highest expression for several sugar transport systems, e.g. for raffinose/stachyose/melibiose, arabinogalactan oligomer/maltooligosaccharide and aldouronate. Moreover, this MAG showed the expression of several glycosidases for the hydrolysis of glycosides, such as glucoamylase (EC 3.2.1.3), cyclomaltodextrinase/neopullulanase (EC 3.2.1.54, 3.2.1.135) and alpha-D-xyloside xylohydrolase (EC 3.2.1.177), which also appeared to be enriched in D3 (Figure 5). Expression of amylases mainly in D3 (Figure 5), e.g., by MAG 262, may be advantageous due to the fact that potato peels were only fed to this digester with a proportion of 20.1% (Table 1). MAG 262 also expressed several key enzymes belonging to the glycolysis (e.g., 2-dehydro-3-deoxyphosphogluconate aldolase, fructose-bisphosphate aldolase, and glucose-6-phosphate isomerase) as well as glycolytic fermentations (pyruvate ferredoxin oxidoreductase, phosphate acetyltransferase, and acetate kinase). Accordingly, the metabolism of MAG 262 mainly relies on the degradation of several single and complex sugars and further fermentation of smaller metabolites; with this, a xylanolytic lifestyle as it was observed for *C. oshimai* could be confirmed for this MAG.

Similar to the description of MAG262, MAG 271 also exhibited high expression of sugar transport systems, e.g. for multiple sugars and aldouronate, but this MAG seemed to be not as versatile regarding the transport of different sugars as MAG 262. Moreover, MAG 271 showed the expression for branched-chain amino acid and peptide/nickel transport systems, the latter one being important for providing nickel as part of the co-factor of NiFe hydrogenases. Among the glycosidases, MAG 271 showed expression for a beta-galactosidase (EC 3.2.1.23) and alpha-L-arabinofuranosidase (EC 3.2.1.55). This MAG also showed expression of glycolytic enzymes, such as L-arabinose isomerase (EC 5.3.1.4), 6-phosphofructokinase (EC 2.7.1.11), fructose-bisphosphate aldolase (EC 4.1.2.13) and glucose-6-phosphate isomerase (EC 5.3.1.9). Moreover, amino acid pathway genes (e.g., *hisB*, *hisD*) were expressed, as well as two peptidases, whereas several other peptidases were encoded in the genome. Interestingly, high expression of 3-isopropylmalate dehydrogenase (*leuB*, EC 1.1.1.85) and 3-isopropylmalate/(R)-2-methylmalate dehydratase small subunit (*leuD*, EC 4.2.1.33 4.2.1.35) was observed for this MAG, which indicates a role during volatile fatty acid (VFA) generation from pyruvate [116]. Here, most probably, isovaleric acid is produced by this MAG, as almost all of the necessary genes (*leuA*, *leuC*, *ilvD*, *ilvC*, *adh*) were present. Moreover, ATP-dependent Clp protease was expressed, which is, as described above, mainly involved in stress response, and a cold shock protein was detected, indicating that MAG 271 has a high temperature optimum similar to the relative *C. oshimai*, which has an optimum temperature at 70 °C. Based on the expression pattern, MAG 271 can be described as an acidogenic member of the order *Caldicoprobacterales*, which is most probably involved in glycolysis and VFA generation from pyruvate.

MAG 267 was assigned to the family *Caldibacillaceae* of the class *Bacilli*, whereby this MAG could not be assigned to a GTDB species cluster and was marked as a taxonomic novelty by the relative evolutionary divergence (RED) method implemented in GTDB-tk [117]. One of the closest relatives of this MAG is *Caldibacillus debilis*${}^{T}$ (DSM 16016; [118]) with an ANI of 70.5%, which shows how distinct this MAG is from known members of the family *Caldibacillaceae*. MAG 267 showed the highest expression regarding the glycolysis (M00001), gluconeogenesis (M00003), Entner–Doudoroff pathway (M00008), citrate cycle (M00009, M00010) and pentose phosphate pathway (M00004) KEGG modules. The highest expressed genes and enzymes of MAG 267 were a mannose phosphotransferase system (PTS EIID, EIIAB), a glycerol dehydrogenase (EC 1.1.1.6), a lysine 6-dehydrogenase (EC 1.4.1.18) and a gentisate 1,2-dioxygenase (EC 1.13.11.4). PTSs catalyze the phosphorylation and transport of carbohydrates and sugars, and are widely spread among *Bacteria* [119]. The glycerol dehydrogenase exhibited the highest proteomic fold change between the three digesters (Section 3.3, Figure 6), with the highest proteomic abundance in digester 3. Expression regarding this enzyme could solely (including all identified MAGs and the unbinned fraction) be identified for MAG 267. Interestingly, the high expression of a gentisate 1,2-dioxygenase by MAG 267 might be related to the degradation of aromatic compounds (e.g., lignin components and aromatic amino acids). This enzyme was also shown for anaerobic organisms, such as *Syntrophus gentianae* [120], and as a functional gene of microbial communities involved in the anaerobic digestion of cotton waste and rice straw [121]. MAG 267 also harbors the complete aromatic degradation KEGG Module (M00569, Catechol meta-cleavage) and several enzymes involved in the degradation of aromatic compounds. While the aerobic degradation pathways of aromatic compounds are well studied, the anaerobic breakdown of these compounds is not yet well resolved. Research on anaerobic *Bacteria* and methanogenic *Archaea* suggested that numerous pathways may be involved in the degradation of aromatic compounds under anoxic circumstances [122]. However, MAG 267 is highly active in glycolysis, as expression was observed for several glycolytic enzymes (e.g., fructose-bisphosphate aldolase, enolase, and pyruvate kinase). As shown above, a lysine 6-dehydrogenase (EC 1.4.1.18) belonged to the highest expressed enzymes of this MAG and, additionally, enzymes involved in amino acid fermentations for lysine, leucine and glutamate were also expressed. Also, key enzymes involved in glycolytic fermentations (formate C-acetyltransferase, acetaldehyde dehydrogenase/alcohol dehydrogenase) as well as acetate oxidation (isocitrate dehydrogenase, aconitate hydratase) were expressed by MAG 267. The species represented by MAG 267 can be classified as an acidogenic/actetogenic member of the family *Caldibacillaceae*, which is mainly active in the glycolysis, amino acid fermentations and glycerol transformations. However, based on the encoded genes and expression, this MAG might also be involved in the degradation of aromatic compounds.

The predominant methanogenic archaeon in digester 3 is represented by MAG 64 assigned to the species *Methanothermobacter wolfeii* (GTDB taxonomy: *Methanothermobacter wolfeii*; NCBI taxonomy: GCA_900095815.1). Relative abundances of this MAG in digesters 1 and 2 are comparatively low (metagenome: D1 0.11%, D2 0.21%; Metaproteome: D1 0.12%, D2 0.26%), whereas the abundance in digester 3 is much higher with 2.52% (metagenome) and 1.61% (metaproteome). It was shown above (Section 3.2.1, Figure 3) that the genus *Methanothermobacter* correlated positively to the process temperature, which is in accordance with the description of this genus as thermophilic methanogens [52]. Moreover, the dominance of the genus *Methanothermobacter* was also shown recently for a thermophilic laboratory-scale digester fed with rice straw and pig manure [123], confirming the adaptation of members of this genus to thermophilic process conditions. The hydrogen-consuming (hydrogenotrophic) methanogen *M. wolfeii* may live in syntrophic association with hydrogen-producing *Bacteria* like members of the family *Syntrophomonadaceae* [124]. Members of the *Syntrophomonadaceae* were also identified within the microbiome of BP35, e.g., MAG 276 was assigned to this family. The complete genome sequence of the *M. wolfeii* str. SIV6, isolated from a full-scale thermophilic biogas digester, as well as its functional potential as revealed by genome-centric metatranscriptomics, was described previously [13]. Comparison of the *M. wolfeii* str. SIV6 isolate with MAG 64 revealed an ANI of 99.76%, demonstrating that the MAG and the isolate are highly related. This finding also proves the fundamental reliability of the reconstruction of this MAG from metagenome sequence data. For MAG 64, the methanogenesis KEGG modules (M00567, max. 43.5%; M00357, max. 34.3%; M00563, max. 21.2%) were the most predominant modules based on the metaproteome. The genes of MAG 64 with the highest expression were *mcrA* (K00399), *mtrH* (K00584) and *hmd* (K13942), which encode the essential enzymes of the hydrogenotrophic methanogenesis pathway. While enzymes encoded by these genes were identified in all three digesters, these were particularly abundant in digester 3. These findings are in great accordance with previously published results of the transcriptional activity of *M. wolfeii* methanogenesis genes under thermophilic conditions [13], as well as the high abundance of methanogenesis enzymes identified in metaproteomes (e.g., [16,125]).

It became obvious that, with eight of the ten described differentially abundant MAGs, the majority of the MAGs could only be classified at higher taxonomic levels (e.g., phylum, order, and family). Moreover, the closest relatives of these MAGs, with ANI values above 99%, were reconstructed MAGs from other biogas metagenomic datasets. Here, a previously published large meta-study from Parks et al. [6], where several metagenomes from databases were assembled and binned to nearly 8000 MAGs, was the basis for the identification of closely related MAGs of the four MAGs 1, 47, 50 and 63. Close relatives of MAG 3 and MAG 144 were also identified in biogas metagenome studies [8,113]. The MAGs 262, 271 and 267, which were identified with higher abundances and expression patterns in digester 3, were not closely related to known genomes or MAGs and could not be assigned to a species cluster within GTDB. This indicates that these MAGs are very likely new members of the order *Caldicoprobacterales* or family *Caldibacillaceae*, which seem to be specialized for specific process conditions, such as higher process temperature or specific feedstocks (e.g., potato peels, high share of sheep or horse manure). Moreover, the detailed description of these MAGs enables insights into the biogas process-related metabolism of these yet unknown members of the order *Caldicoprobacterales* and family *Caldibacillaceae*. Only MAGs 50 and 64 could be taxonomically assigned on the genus or species level. Nevertheless, based on ANI, the closest relative of MAG 50 was also a MAG, which was binned before by Parks et al. [6] and is not an isolate of the genus *Herbivorax*. The archaeal MAG 64 was the only MAG which could directly be related to the completely sequenced and analyzed isolate *M. wolfeii* SIV6. This shows how valuable and important metagenomic binning approaches are for the elucidation of the still unknown microbial “dark matter” of biogas microbiomes.

The combined analysis of the genetic potential and expressed functions of ten differentially abundant MAGs of the three digesters allowed a detailed characterization of their metabolic functions, outlining their potential roles in the biogas process (Figure 8). As hypothesized, these MAGs may represent specialized taxa which seem to be adapted to the different process conditions (e.g., process temperatures and feedstocks) of the three digesters of BP35. Even though in this study only 10 differentially abundant MAGs were analyzed in detail regarding their biogas-related metabolic functions, nevertheless, as hypothesized, potential substitutions of the functions of MAGs in one of the digesters by other MAGs in the other digesters could be found. For example, MAG 47 was characterized as a proteolytic amino acid degrader in D1, whereas MAG 1 might fulfill this proteolytic function in D2. MAGs 50 and 63 showed expression regarding xylose degradation mainly in D1, whereas MAGs 1 and 144 showed this mainly in D2. In addition, functions were identified that were specific for only one of the three digesters. Here, the expression of a glycerol dehydrogenase in D3 (Figure 6) by MAG 267 seems to have a significant role only in this digester.

#### 3.4.2. High-Quality MAGs with Similar Relative Abundances and Metabolic Activities in the Three Digesters Indicate Their Resilience and Importance for a Stable Biogas Process

The bacterial MAGs 80, 160, 162 and the archaeal MAG 246 showed more even distributions in the three digesters, which indicates their resilience to different process conditions. It was hypothesized and shown that resilient taxa support a stable microbiome in biogas digesters and thus a stable anaerobic digestion process since they do not respond sensitively to changes of the process conditions (e.g., [1,2,3]). In order to identify the role of these MAGs in the digesters of BP35, their metabolic functions regarding expressed KOs and KEGG modules (Table S4), as well as key genes and enzymes related to the anaerobic digestion process were analyzed.

**Figure 9:**
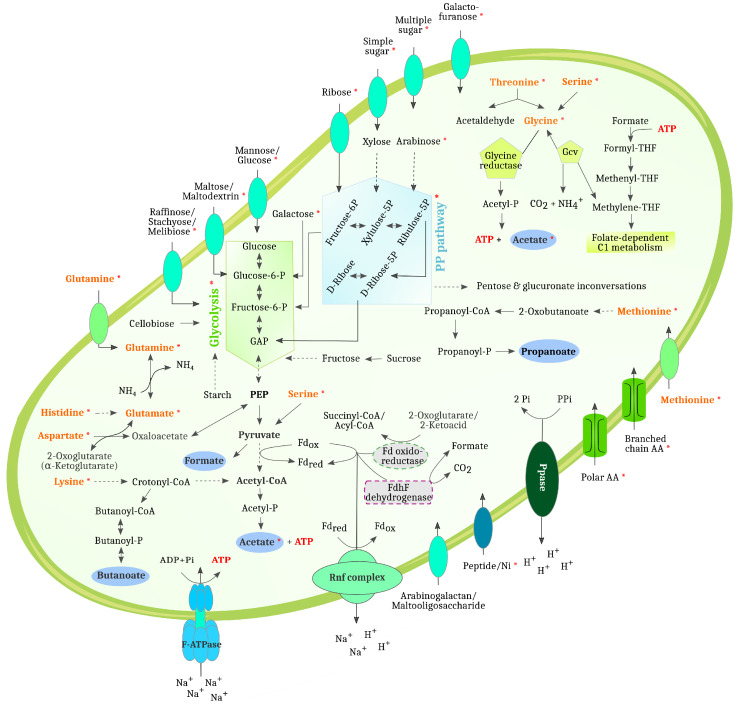
Overview of the main metabolic pathways of MAG 80. Amino acids were marked in orange. Metabolites and pathways were marked with an red asterisk when, for corresponding enzymes, expression was observed based on MAG-centric metaproteomics. CoA = coenzyme A, Fd = ferredoxin, GAP = glyceraldehyde 3-phosphate, Gcv = glycine cleavage system, PEP = phosphoenolpyruvate, PP pathway = pentose phosphate pathway, PPi = pyrophosphate, Rnf = ferredoxin:NAD${}^{+}$ oxidoreductase.

MAG 80 is the most abundant organism in the three digesters, with metagenome-based relative abundances of 11.55%, 17.79% and 15.71%, and metaproteomic relative abundances of 22.24%, 25.15% and 20.30% in digesters 1, 2 and 3, respectively. MAG 80 was assigned to the class *Limnochordia* of the phylum *Firmicutes* (GTDB: DTU010 sp002391385; NCBI: GCA_002391385.1), and belongs to a new species cluster within GTDB. The closest related MAG UBA3914 (GCA_002391385.1, ANI: 99.9%) was assembled before by Parks et al. [6] from an anaerobic digester metagenome. Another closely related MAG AS24abBPME_73 (GCA_012523675.1, ANI 99.9%), which also belongs to the new GTDB species cluster, was assembled by Campanaro et al. [8]. This MAG was also identified in recent metagenome-based biogas studies [126,127,128,129], where it was named differentially (DTU-pt_99, DTU-pt_144, Limnochordia sp. GSMM97) and described as potential homo-acetogenic bacterium, with potential syntrophic interactions with *Methanothermobacter wolfeii*. Thus, MAG 80 may also have potential syntrophic interactions with the archaeal MAG 64, which was taxonomically assigned as *M. wolfeii*. It became obvious that this MAG may have an important role not only within the biogas digesters analyzed in this study but also in biogas microbiomes in general. Based on the relative abundance of the proteomic spectral counts, several KEGG modules were highly expressed by this MAG. Among these modules were those for glycolysis (M00001), which describes the glucose to pyruvate pathway, gluconeogenesis (M00003) from oxaloacetate to fructose-6P, glycogen degradation (M00855), describing the glycogen to glucose-6P pathway, the semi-phosphorylative Entner–Doudoroff pathway (M00308) from gluconate to glycerate-3P, and the pyruvate oxidation pathway (M00307), which describes the pyruvate to acetyl-CoA reactions. The highest expressed enzymes of MAG 80 represent several transport systems supporting the transport of peptides/nickel (K02035), maltose/maltodextrin (K10108), raffinose/stachyose/melibiose (K10117), multiple sugars (K02027), simple sugars (K02058) glucose/mannose (K17315) and branched-chain (K01999) as well as polar amino acids (K02030) (Figure 9). Besides the transport systems, also enzymes like PulA (K01200) for the hydrolysis of pullulan and starch, NifJ (K03737), a pyruvate-ferredoxin/flavodoxin oxidoreductase, GapA (K00134), a glyceraldehyde 3-phosphate dehydrogenase and AtoB (K00626), an acetyl-CoA C-acetyltransferase featured high abundances based on the proteome.

**Figure 10:**
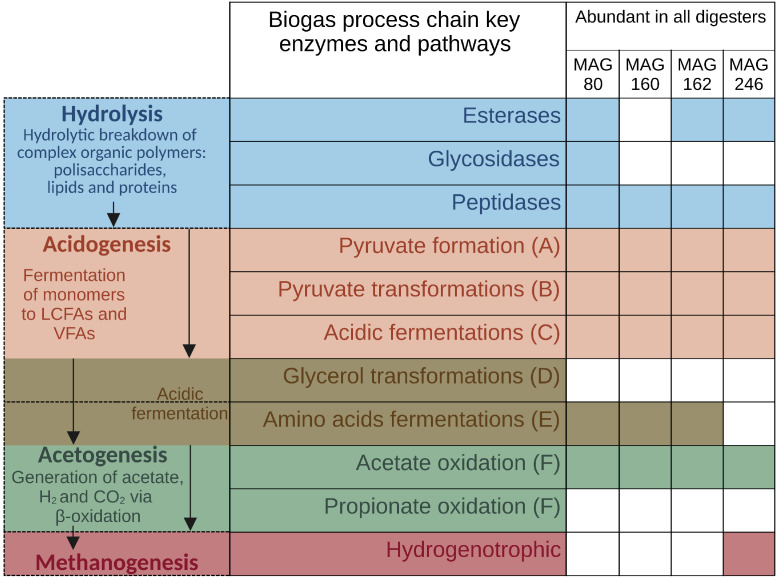
Proteomic expression of biogas process chain key enzymes and pathways for four in the three biogas digesters’ evenly abundant MAGs. Key enzymes and pathways, as well as the categorization (A–F) were derived from Sikora et al. [43] and were marked when one key enzyme was present based on the metaproteome counts.

The expression pattern of the biogas-related key genes and enzymes revealed a versatile metabolism of MAG 80 (Figure 9 and Figure 10). Most of these enzymes belong to the hydrolysis, glycolysis and glycolytic fermentations. Moreover, the complete glycine reductase pathway was expressed by this MAG, which belongs to the fermentation of amino acids within the acidogenesis (Figure 9). Key enzymes belonging to the oxidation of acetate, butyrate and ethanol within the acetogenesis were also expressed by this MAG, but the corresponding pathways remain incomplete based on the genomic potential of this MAG. This observation might be explained by the fact that this MAG has a completeness of about 83% and a contamination of 3%, which might result in the prediction of incomplete metabolic pathways.

Based on the genome and proteome analyses, MAG 80 has the capability to use several carbohydrates and amino acids as substrates, producing mainly acetate as the end product (Figure 9). MAG 80 was shown to be metabolically versatile and ubiquitously active in many pathways of the biogas process chain, and seems to be resilient to the different conditions within the three digesters.

MAG 160 was assigned to the phylum *Firmicutes*_G and is colesly related to a MAG that was also binned by Parks et al. [6] from an anaerobic digester metagenome (GTDB: UBA4971 sp900019985; NCBI: GCA_900019985.1; ANI: 99.7%). MAG 160 showed metagenomic abundances between 3.18 and 4.53% and metaproteomic abundances between 2.50 and 3.8% in the three digesters. For this MAG, metaproteomic data revealed three highly expressed KEGG modules. These are pyruvate oxidation (M00307), C1-unit interconversion (M00140) and cysteine biosynthesis (M00021). The highest expressed enzymes of MAG 160 belong to transport systems for peptide/nickel (K02035), multiple sugar (K02027), simple sugar (K02058), tungstate (K05772), and iron (K02016) as well as a 2-hydroxycyclohexanecarboxyl-CoA dehydrogenase (K07535), which might be involved in the anaerobic degradation of aromatic compounds [130]. Among the biogas key enzymes, expression was observed for a hydrolytic serine protease, as well as the glycolytic enzymes phosphoglycerate kinase, 6-phosphofructokinase and fructose-bisphosphate aldolase. Also, key enzymes involved in glycolytic, acidic and amino acid fermentations were expressed by this MAG (pyruvate ferredoxin oxidoreductase, phosphate acetyltransferase, fumarate hydratase, methylenetetrahydrofolate dehydrogenase, and glutamate dehydrogenase). Here especially, the pyruvate ferredoxin oxidoreductase (EC 1.2.7.1) belongs to the highest expressed enzymes of this MAG. Moreover, the 2-oxoglutarate/2-oxoacid ferredoxin oxidoreductase was also expressed by MAG 160, indicating a possible role in acetate oxidation. Besides the biogas-related functions of this MAG, expression for the Clp and HsIUV protease was observed, which might be involved in the stress response as discussed above. However, due to the overall expression pattern, this MAG seems to be mainly involved in the glycolysis and glycolytic fermentations within acidogenesis (Figure 10).

MAG 162 was assigned to the genus *Acetomicrobium* (GTDB: *Acetomicrobium* sp012518015; NCBI: GCA_012518015.1, ANI: 99.4%) and showed metagenomic relative abundances of 1.14–2.22% and metaproteomic abundances of 1.24–1.58%, whereby slightly lower abundances were found in D3 and higher abundances in D2. The highly similar MAG AS24abBPME_148 (GCA_012518015.1) was previously reconstructed from an anaerobic digester metagenome by Campanaro et al. [8] and Gaspari et al. [126]. The highest expressed KEGG modules of this MAG were the propanoyl-CoA metabolism (M00741), followed by pyruvate oxidation (M00307) and histidine degradation (M00045). The expression of the first two modules was more evenly distributed between the three digesters, whereas the latter module had its highest expression in D3 and was not expressed in D1. The highest-expressed gene products of this MAG were chaperonin (K04077), bacterioferritin (K03594), a branched-chain amino acid transport system (K01999), and glycerol kinase (K00864). The expression pattern of the biogas-related key enzymes revealed that MAG 162 expressed all enzymes involved in amino acid fermentations via the Stickland reaction to acetate (Figure 10). The key enzymes alanine dehydrogenase, leucine dehydrogenase, glycine reductase as well as the acetate kinase were among the highest-expressed enzymes of this MAG. Besides the key enzymes, also proteomic spectral counts for transport systems for amino acids, like those for branched-chain and polar amino acids, were identified within this MAG, which underlines the capability of this MAG to ferment amino acids. However, while single enzymes involved in glycolytic fermentations (e.g., pyruvate ferredoxin oxidoreductase, and acetyl-CoA acetyltransferase) as well as acetate and propionate oxidation (e.g., 2-oxoglutarate/2-oxoacid ferredoxin oxidoreductase, and methylmalonyl-CoA/ethylmalonyl-CoA epimerase) were expressed by this MAG, the corresponding pathways remain incomplete based on the genomic potential of this MAG. It was shown above that the genus *Acetomicrobium* exhibited correlations with the concentrations of total VFAs and acetic acid (Section 3.2.1, Figure 3) within samples from the digesters of this biogas plant. This seems reasonable since the genus and also the genomic and proteomic analyses of MAG 162 indicated the acetate-producing capability of these organisms.

The archaeal MAG 246 was assigned to the species *Methanoculleus thermohydrogenotrophicum* (GTDB: *Methanoculleus thermohydrogenotrophicum*, NCBI: GCA_001512375.1, ANI: 99.6%). This MAG showed low metagenome-based relative abundances between 0.02 and 0.28%, whereas the metaproteomic relative abundance was much higher with values ranging from 2.65 up to 11.03%. However, as already addressed in Section 3.4, the methanogenesis-related enzymes were reported to be among the most abundant metaproteins [16,125], and the genomic abundances of methanogenic *Archaea* and their metabolic activity can differ significantly [15,91]. For this MAG, the methanogenesis KEGG modules (M00567, max. 69.8%; M00357, max. 41.5%; M00356, max. 38.4%) were the highest expressed modules based on the metaproteome. The highest-expressed enzymes Mer (K00320), McrB (K00401), McrA (K00399) and McrG (K00402) belong to the hydrogenotrophic methanogenesis pathway. The key enzymes for methanogenesis as described by Sikora et al. [43] were mostly present and abundant for this MAG based on their proteomic abundances. The Hmd, Cdh (acetotrophic methanogenesis or Wood–Ljungdahl-pathway) and MtaB (methylotrophic methanogenesis) key enzymes were missing for this MAG, which is reasonable since the species *Methanoculleus thermohydrogenotrophicum* is not known for performing acetotrophic or methylotrophic methanogenesis.

The four MAGs showing high abundances across all three digesters were assigned on the phylum, class, genus and species levels, whereby MAG 80, which was the most abundant and active MAG in all three digesters, was only taxonomically assigned on the class level. The most closely related MAGs, with ANI values greater than 99%, of MAG 80 and 160 originated from a meta-study by Parks et al. [6], as also shown above for four of the differentially abundant MAGs. Closely related MAGs of MAG 162 and 80 were identified also in other metagenomic biogas studies (MAG 162: [8,126]; MAG 80: [8,126,127,128,129]. These findings again demonstrate the importance of metagenome-based research in the biogas field to unravel and characterize still unknown organisms within biogas microbiomes.

In summary, the detailed metagenome and metaproteome analysis of the four evenly distributed MAGs in the three digesters enabled the metabolic characterization of the functional potential as well as the actually expressed functions of these organisms, as well as identifying their roles in the biogas process (Figure 10). Here, especially MAG 80 could have an important role within biogas microbiomes, as it has a versatile metabolism and was shown to be ubiquitously abundant and active in the three digesters of BP35 and was also identified in several other biogas studies. Such versatile and resilient microorganisms are of immense value in industrial processes, especially in the context of biogas production, as these do not respond sensitively to changing or different process conditions.

As hypothesized, MAGs were identified that showed more even abundances and constant metabolic activities in all three digesters, although these digesters were operated differently. Thus, these MAGs appeared to be more resilient to the different process conditions, which could indicate their important role for stable process conditions in biogas digesters.

## 4. Conclusions

The combination of MAG-centric metagenomics and metaproteomics enabled the analysis of the metabolic functional potential and the expressed functions of reconstructed MAGs and also of the whole microbial community, including the currently uncultivable microbial “dark matter” from the three digesters of BP35. MAGs adapted to the different biogas digester conditions, such as increased process temperatures. Specific input materials were identified, and their metabolic functions within the biogas process chain were able to be deduced.

Moreover, resilient MAGs whose metagenomic and metaproteomic abundances appeared to be independent of the digester process conditions were observed. Based on the knowledge about the metabolism of the analyzed MAGs, cultivation-based methods could be further improved in order to isolate previously uncultivable microorganisms from biogas digesters. Furthermore, the results contribute to establishing the basis for future microbiome-based management strategies for biogas digesters. Here, the development of specific inocula appears to be a particularly promising approach. Successful bioaugmentation using a bacterial inoculum was previously demonstrated for reactors set up for fermentative biohydrogen production [131]. For inoculum composition, resilient taxa could be considered when varying process conditions (e.g., diverse input materials and varying process temperatures) are expected. On the other hand, specialized taxa can be included when biogas digesters are operated under constant conditions regarding temperature or specific substrates.

Although the microbial communities of the three digesters of BP35 were shown to be different from the other biogas microbiomes taxonomically profiled in a previous study, isolates or closely related MAGs from previously metagenomically analyzed biogas digesters were able to be identified for almost all of the MAGs described in detail in this study. This indicates that the identified MAGs might also play important roles in other biogas microbiomes. It furthermore demonstrates how the deposition of metagenomes and MAGs in databases enables better characterization of biogas metagenomes and metaproteomes, as well as understanding of biogas microbiomes and their metabolic functions. This provides the basis to develop microbiome-based management strategies for the monitoring and process control of biogas digesters in the future.

MAG-centered metagenome studies are clearly limited to the more abundant microbiome members since genomes of low-abundance or even rare microorganisms will not be assembled and binned. This shortcoming can be addressed by deeper metagenome sequencing or the implementation of long-read sequencing technologies. Moreover, the consideration of other omics-technologies, such as metatranscriptomics and metabolomics, would provide insights at all levels of the information flow from the genome sequence to metabolism. Integrative analysis of all omics-data in combination with corresponding metadata will allow the reconstruction of metabolic networks, identification of cooperating sub-microbiome assemblages and dependencies and interactions between biogas microbiome members.

## Figures and Tables

**Table 1:** Mean values of the main process parameters of the three digesters of biogas plant 35. Percentages of the input materials are given as the share of fresh matter of the feed mixture, % (m/m). OLR = Organic Loading Rate, HRT = Hydraulic Retention Time, VS = volatile solids.

Biogas Digester	Maize Silage [%]	Grass Silage [%]	Cereals [%]	Solid Manure (Sheep, Cattle) [%]	Chicken Manure [%]	Potato Peels [%]	OLR [kg_VS_ m${}^{-3}$d${}^{-1}$]	HRT [d]	Temperature [°C]
1	34.00	27.85	6.99	25.38	5.8	-	4.53	71	44.5
2	36.20	29.45	7.39	21.96	5.0	-	4.32	75	50.0
3	-	20.55	-	59.32	-	20.13	0.41	475	56.3

**Table 2:** Metagenome-based relative abundance and fraction of assigned metaproteins of unbinned contigs and fraction of metagenomically assembled genomes (MAGs) with low quality (below 50% completeness and/or above 10% contamination), as well as the fraction of high-quality (HQ) MAGs below 0.5% relative abundance and above 0.5% relative abundance either in the metagenome or metaproteome.

	Contigs, Low Quality and HQ MAGs	Digester 1	Digester 2	Digester 3
Metagenome- based relative abundance	Unbinned contigs and low quality MAGs	42.02%	37.02%	37.81%
	HQ MAGs < 0.5% relative abundance	11.77%	6.48%	5.85%
	HQ MAGs > 0.5% relative abundance	46.21%	56.50%	56.34%
Fraction of assigned metaproteins	Unbinned contigs and low quality MAGs	35.03%	36.06%	44.19%
	HQ MAGs < 0.5% relative abundance	6.92%	3.98%	3.69%
	HQ MAGs > 0.5% relative abundance	58.05%	59.96%	52.12%

## Data Availability

Metagenome read data, assembled contigs and bins of the 46 high quality MAGs were submitted to the European Nucleotide Archive (ENA) and are archived under the study accession number PRJEB39821. Raw reads are available under accession numbers ERS15412147 through ERS15412155. The assembly has the accession number ERZ18327520, accessions of the individual biological samples are ERS15412147–ERS15412155. Accessions for individual MAG bins can be found in supplementary Table S2. The mass spectrometry proteomics data have been deposited to the ProteomeXchange Consortium via the PRIDE partner repository with the dataset identifier PXD044571.

## Supplementary Materials

The following are available online at https://www.mdpi.com/article/10.3390/microorganisms11102412/s1, Table S1: Process and chemical analysis parameters of the three differentially operated digesters of biogas plant 35. Table S2: Metagenomically assembled genome (MAG) overview. Including genome size, completeness, contamination, taxonomic assignment based on GTDB, metagenomic and metaproteomic abundances in the three digesters of biogas plant 35, as well as the European Nucleotide Archive (ENA) accession numbers. Table S3: KEGG orthologies and modules with the highest proteomic abundances for ten MAGs, which were differentially abundant in the three digesters of biogas plant 35. Proteomic abundance is ranked based on the sum of normalized spectral counts assigned to the respective orthology/module. Numbers in square brackets indicate in which digester the count number was detected. Table S4: KEGG orthologies and modules with the highest proteomic abundances for the four MAGs that were equally abundant in the three digesters of biogas plant 35. Proteomic abundance is ranked based on the sum of normalized spectral counts assigned to the respective orthology/module. The number in square brackets indicates the digester in which the highest count was detected.
